# Hydrophobicity‐Controlled Self‐Assembly of Supramolecular Peptide Nanotubes in Water

**DOI:** 10.1002/anie.202423828

**Published:** 2025-04-14

**Authors:** Min Zeng, William Parsons, Yixuan Chen, David K. Chalmers, Sébastien Perrier

**Affiliations:** ^1^ Department of Chemistry University of Warwick Coventry CV4 7AL UK; ^2^ Monash Institute of Pharmaceutical Sciences Monash University Parkville Victoria 3052 Australia; ^3^ Warwick Medical School University of Warwick Coventry CV4 7AL UK

**Keywords:** Cyclic peptide, Molecular dynamics simulation, Nanotube, Self‐assembly, Supramolecular

## Abstract

Polymer‐conjugated peptides are attractive building blocks for the construction of new nanomaterials. However, the ability to control the self‐assembly of these materials remains a major limitation to their wider utilization. Herein, we report a facile strategy to fine‐tune the assembly of water‐soluble hydrophilic polymer‐conjugated cyclic peptides by incorporating a defined, short hydrocarbon linker between the polymer and peptide. This addition creates a well‐defined hydrophobic “inner shell” that suppresses water from disrupting the organized peptide hydrogen bond network. Our approach is demonstrated using a series of cyclic peptide‐linker‐PDMA conjugates that were evaluated by asymmetric flow field flow fractionation, small angle neutron scattering and transmission electron microscopy. Molecular dynamics simulations were also used to show how the polymer and the peptide stacks interact and illustrate the impact of this hydrophobic inner shell approach. This strategy provides a modular approach to fine control the nanotube self‐assembling behavior. We expect that this technique will improve the versatility of peptide nanotubes for the engineering of advanced nanomaterials.

## Graphical abstract

Cyclic peptide–polymer conjugates, with varied length of hydrophobic alkyl linker group, are synthesized and assembled into nanotubes in water. Both synthetic studies and computational molecular dynamics simulations confirm the degree of hydrophobicity, providing an avenue to tune the length and dynamic nature of the peptide nanotubes.

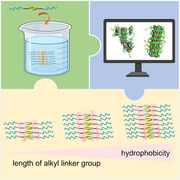

## Introduction

Harnessing the ability of peptide to self‐assemble into large, complex structures is promising route to the engineering of new functional nanomaterials. Among the wide range of known self‐assembling peptides, head‐to‐tail cyclic peptides composed of alternating d‐ and l‐α‐amino acids (CPs) have emerged as versatile modules for creating peptide nanostructures.^[^ ^1^, ^2^, ^3^, ^4^, ^5^, ^6^, ^7^, ^8^, ^9^, ^10^, ^11^, ^12^ ^]^ These peptides form a flat structure with the amide groups oriented perpendicular to the plane of the ring that enables self‐assembly through intramolecular hydrogen boding.^[^ ^5^ ^]^ The CP unimers assemble to form peptide nanotubes that have great potential in biologically relevant applications, such as artificial ion channels,^[^ ^13^, ^14^ ^]^ for drug delivery,^[^ ^15^, ^16^, ^17^, ^18^, ^19^ ^]^ as antibacterial materials,^[^ ^20^ ^]^ or as biosensors.^[^ ^21^, ^22^, ^23^ ^]^ However, despite progress in the design of more complex and functionalized cyclic peptide building blocks (unimers), it remains a key challenge to exert control over the degree of self‐assembly. The propensity of CP unimers to assemble directly controls the size of resulting nanotubes. Therefore, to design nanotube materials with well‐defined physicochemical properties, we must be able to control the process of self‐assembly.

The stability of assembled CP nanotubes depends on many factors, including the amino acid sequence of the CP unimer itself or attachment of pendant groups such as polymers. Silk et al. have shown that alignment of both oppositely charged as well as hydrophobic residues within the CP core of the nanotube can promote stability in an aqueous environment.^[^ ^6^ ^]^ Covalently tethering CPs offers alternative strategy to control unimer assembly.^[^ ^24^, ^25^, ^26^, ^27^ ^]^ Attaching polymers to the periphery of the cyclic peptides is also a powerful technique to modulate supramolecular assembly and solubility.^[^ ^28^, ^29^, ^30^, ^31^, ^32^, ^33^, ^34^ ^]^ Conjugation of a hydrophilic polymer to the cyclic peptide generates water‐soluble nanotubes.^[^ ^35^ ^]^ Asymmetric conjugation of hydrophilic and hydrophobic homopolymer arms can create nanotubes that undergo secondary self‐assembly, forming hierarchical superstructures termed tubisomes,^[^ ^36^, ^37^ ^]^ which have good biocompatibility and can be used as drug delivery vectors with a high drug loading content.^[^ ^38^ ^]^ Providing an additional level of control over the nanotube assembly, amphiphilic diblock copolymers have been used to create conjugates with a hydrophobic inner layer and a hydrophilic outer layer.^[^ ^31^ ^]^ Compared to cyclic peptides conjugated with a hydrophilic homopolymer, amphiphilic diblock copolymer conjugates are more stable and have less dynamic behavior.

**Scheme 1:**
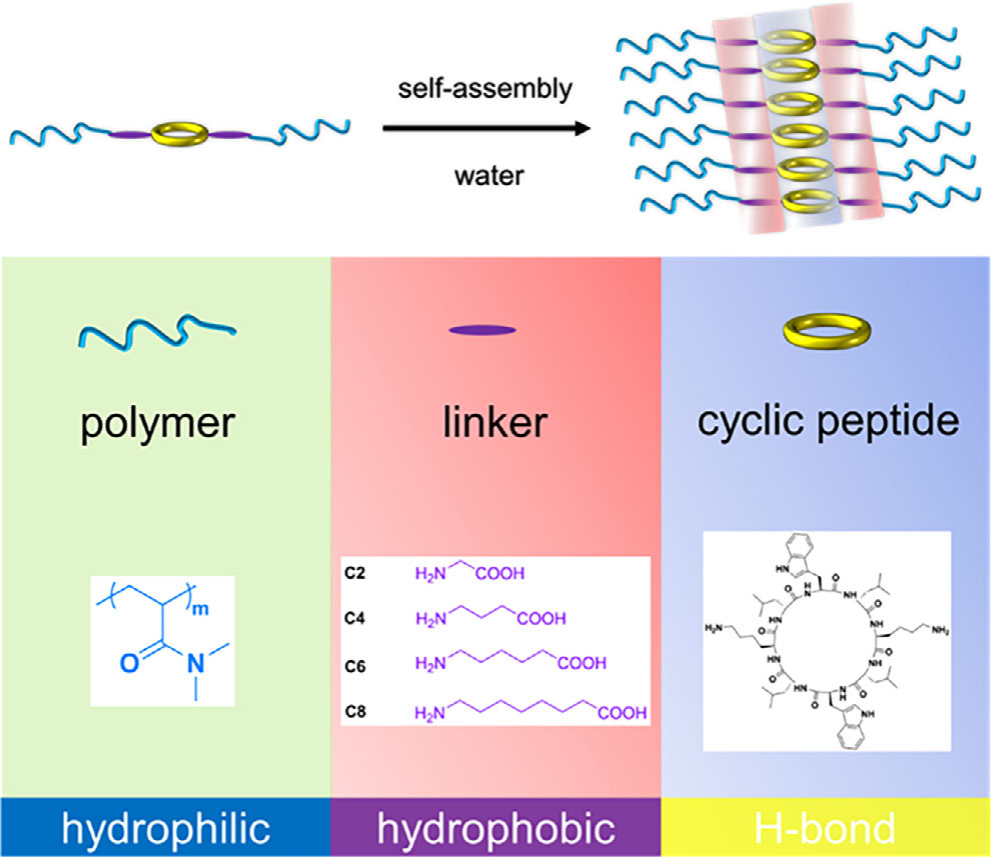
Incorporation of a short hydrophobic linker domain into cyclic peptide–polymer conjugates to achieve fine control of nanotube assembly in water.

In this work, we envisioned that incorporating a short hydrophobic section between the CP unimer and an attached hydrophilic polymer could be used to achieve fine control of nanotube assembly (Scheme 1). Evaluating this proposition, we have synthesized a series of cyclic peptide–polymer conjugates that incorporate hydrocarbon linkers with 2, 4, 6, and 8 carbon atoms between the peptide and hydrophilic poly(*N*,*N*‐dimethylacrylamide) PDMA. We have characterized the propensity of the conjugated unimers to form nanotubes in water by asymmetric flow field flow fractionation (AF_4_), small angle neutron scattering (SANS), and transmission electron microscope (TEM). Finally, we have used molecular dynamics simulations to elucidate the role of the hydrophobic linker and polymer chains in stabilizing the nanotubes.

## Results and Discussion

### Synthesis and Characterization of Cyclic Peptide–Polymer Conjugate

The core cyclic peptide, cyclo[(l‐Lys‐d‐Leu‐l‐Trp‐d‐Leu)_2_], which contains two free amine groups, was synthesized by solid phase peptide synthesis followed by cyclization and deprotection (Scheme S5). Considering the position of the linker, a “grafting to” method was employed to synthesize the cyclic peptide–polymer conjugate. Hydrophilic PDMA polymer was synthesized by RAFT solution polymerization of DMA in 1,4‐dioxane at 70 °C using *N*‐hydroxysuccinimide‐(propanoic acid)yl butyl trithiocarbonate (PABTC‐NHS, Scheme S3) as the chain transfer agent. After 2 h of polymerization, the monomer conversion reached 99% as determined by proton nuclear magnetic resonance (^1^H NMR). The degree of polymerization (DP) of PDMA was calculated as 45 from the ^1^H NMR spectrum (Figure S6), and the calculated molecular weight was *M*_n,NMR_ = 4700 g mol^−1^. The size exclusion chromatography (SEC) molecular weight distribution showed a unimodal peak and a narrow dispersity (Figure S7, *M*_n,SEC_ = 4700 g mol^−1^ and *Đ* = 1.18), which confirms a good control of RAFT polymerization.

The NHS terminal group of the PDMA‐NHS was modified with a series of linkers (Scheme S4): glycine (C2), 4‐aminobutyric acid (C4), 6‐aminocaproic acid (C6), and 8‐aminocaprylic acid (C8). The reactivity of the NHS group at the end of PDMA chain favors reaction with the free amine of the linker, resulting in an amide bond formation with the functionalized polymer. ^1^H NMR spectrum confirms that the C6 molecule was successfully attached to end of polymer chain, with an end group functionality measured as 98.5% (Figure S10). Using the same method, we synthesized a series of PDMA polymers bearing different length linkers, including PDMA‐C2, PDMA‐C4, PDMA‐C6, and PDMA‐C8 (Figures S8–S12). The *M*_n_ for PDMA‐C2, PDMA‐C4, PDMA‐C6, and PDMA‐C8 was 4000, 4200, 4300, and 4500 g mol^−1^, respectively (Table 1). The free carboxylic acid present in the linker was reacted with lysine sidechain amines on the cyclic peptide to form the cyclic peptide–polymer conjugates.

**Table 1:** Molecular weight and dispersity of PDMA polymers and cyclic peptide–polymer conjugates.

Sample	*M*_n, SEC_ (g mol^−1^)^a)^	*Ð* ^a)^
PDMA‐NHS	4700	1.18
PDMA‐C2	4000	1.23
PDMA‐C4	4200	1.20
PDMA‐C6	4300	1.21
PDMA‐C8	4500	1.20
(PDMA‐C2)_2_‐CP	12 500	1.24
(PDMA‐C4)_2_‐CP	11 200	1.30
(PDMA‐C6)_2_‐CP	11 800	1.25
(PDMA‐C8)_2_‐CP	12 600	1.25

^a)^ Measured by DMF SEC.

**Figure 1:**
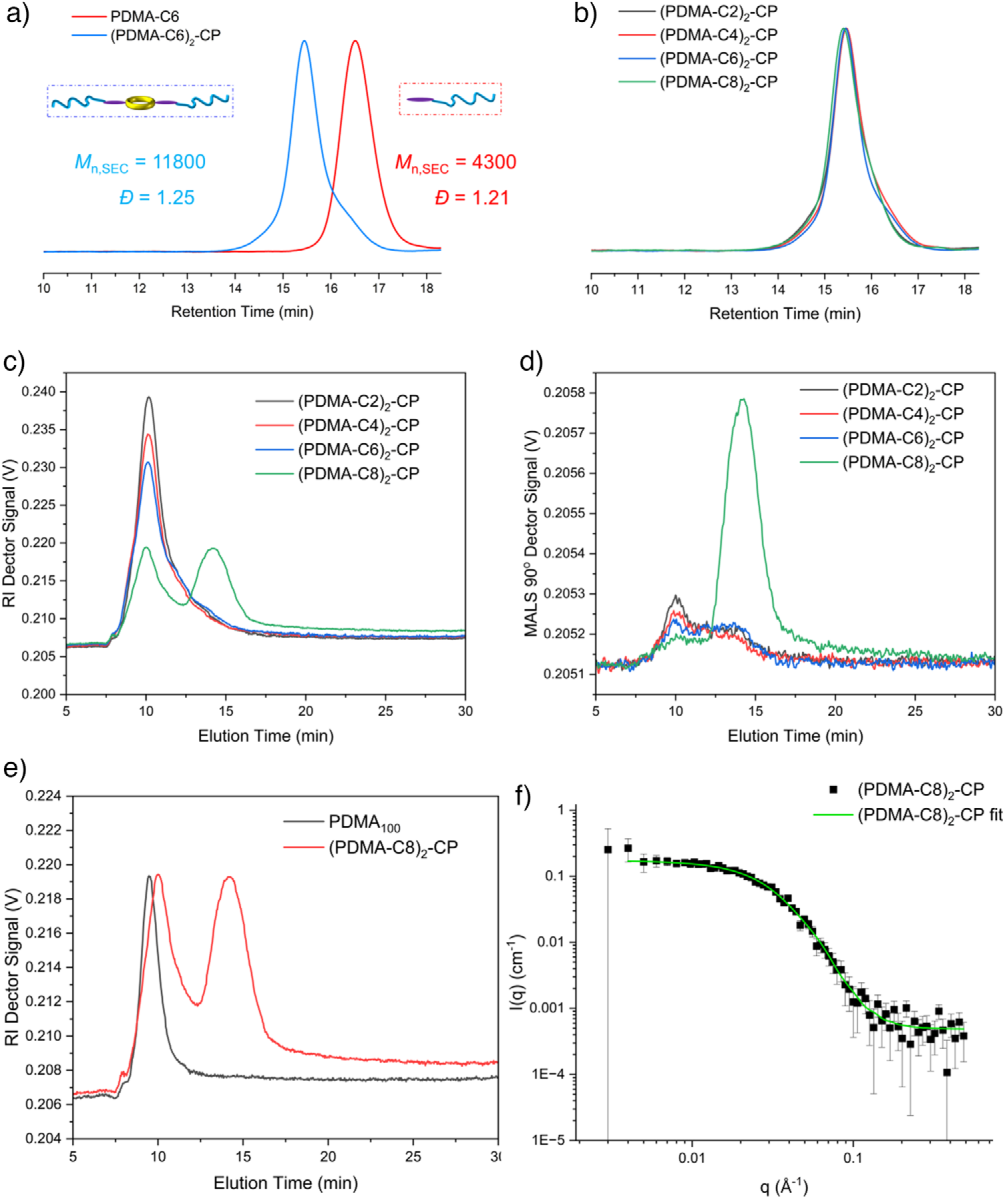
a) Typical overlayed SEC traces of PDMA‐linker and (PDMA‐linker)_2_‐CP, using C6 as an example linker. b) Overlayed SEC traces of (PDMA‐C*_x_*)_2_‐CP (*x* = 2, 4, 6, 8). c) Overlayed AF_4_‐RI traces of (PDMA‐C*_x_*)_2_‐CP (*x* = 2, 4, 6, 8). d) Overlayed AF_4_‐MALS traces of (PDMA‐C*_x_*)_2_‐CP (*x* = 2, 4, 6, 8). e) Overlayed AF_4_‐RI traces of (PDMA‐C8)_2_‐CP and linear homopolymer PDMA_100_. f) SANS plot of (PDMA‐C8)_2_‐CP (black) and fitted to a core–shell cylinder model (green) using SasView software.

The peptide amines were coupled to the polymer carboxylic acid group using 1‐[bis(dimethylamino)methylene]‐1*H*‐1,2,3‐triazolo[4,5‐b]pyridinium 3‐oxid hexafluorophosphate (HATU), with *N*,*N*‐diisopropylethylamine (DIPEA) as a base, and the excess free polymer was removed by centrifugal filtration. The resulting two‐armed conjugate (PDMA‐C6)_2_‐CP showed a left‐shifted molecular weight distribution on SEC and the double molecular weight of the polymer (Figure 1a). The *M*_n_ of the polymer–linker (PDMA‐C6) alone was 4300 g mol^−1^ and the conjugate was 11 800 g mol^−1^ (Table 1). The conjugates with C2, C4, and C8 were prepared following the same method (Figures 1b and S13–S15). All cyclic peptide–polymer conjugates exhibited a narrow dispersity (*Đ* ≤ 1.30, Table 1).

### Hydrophobicity‐Controlled Self‐Assembly of Cyclic Peptide Nanotubes

The self‐assembly of the conjugates was analyzed by AF_4_ at a concentration of 1 mg mL^−1^ in water. AF_4_ separates analytes in situ by hydrodynamic size using a thin open flow channel and is a powerful analytical technique for supramolecular systems.^[^ ^39^, ^40^, ^41^ ^]^ The refractive index (RI) fractogram for the (PDMA‐C2)_2_‐CP, (PDMA‐C4)_2_‐CP, and (PDMA‐C6)_2_‐CP conjugates (Figure 1c) shows a single peak at elution time of 10 min, whereas two peaks are observed at 10 and 15 min for the (PDMA‐C8)_2_‐CP conjugate, suggesting the length of hydrophobic alkyl group affects the self‐assembly behavior of the conjugates.

As a nonassembling control, we prepared a linear homopolymer PDMA with a DP of 100 (*M*_n,SEC_ = 10 500, *Đ* = 1.17, Figure S16). Analyzing this PDMA_100_ by AF_4_ showed a peak at 10 min, which overlapped with the low elution time peak of the conjugate (Figure 1e), thus confirming that the peak with elution time at 10 min in the conjugates is associated with unimers. A multi angle light scattering (MALS) detector was also used as complementary detector and revealed no peak at 10 min, confirming these are not assembled, whereas a peak at 15 min was observed for the (PDMA‐C8)_2_‐CP conjugate, suggesting the formation of supramolecular assemblies in this case (Figure 1d).

The formation of nanotube assemblies was confirmed using SANS. The scattering plot of the (PDMA‐C8)_2_‐CP was best fitted to a core–shell cylinder model (Figure 1f and Table S1). We, therefore, conclude that the (PDMA‐C2)_2_‐CP, (PDMA‐C4)_2_‐CP, and (PDMA‐C6)_2_‐CP conjugates exist in water as unimers, whereas the (PDMA‐C8)_2_‐CP conjugate is present as both unimers and nanotubes. The length of the hydrophobic linker, therefore, affects the self‐assembly of the cyclic peptide nanotubes. The dynamic nature of these self‐assemblies is consistent with previous work in our group.^[^ ^35^ ^]^

**Figure 2:**
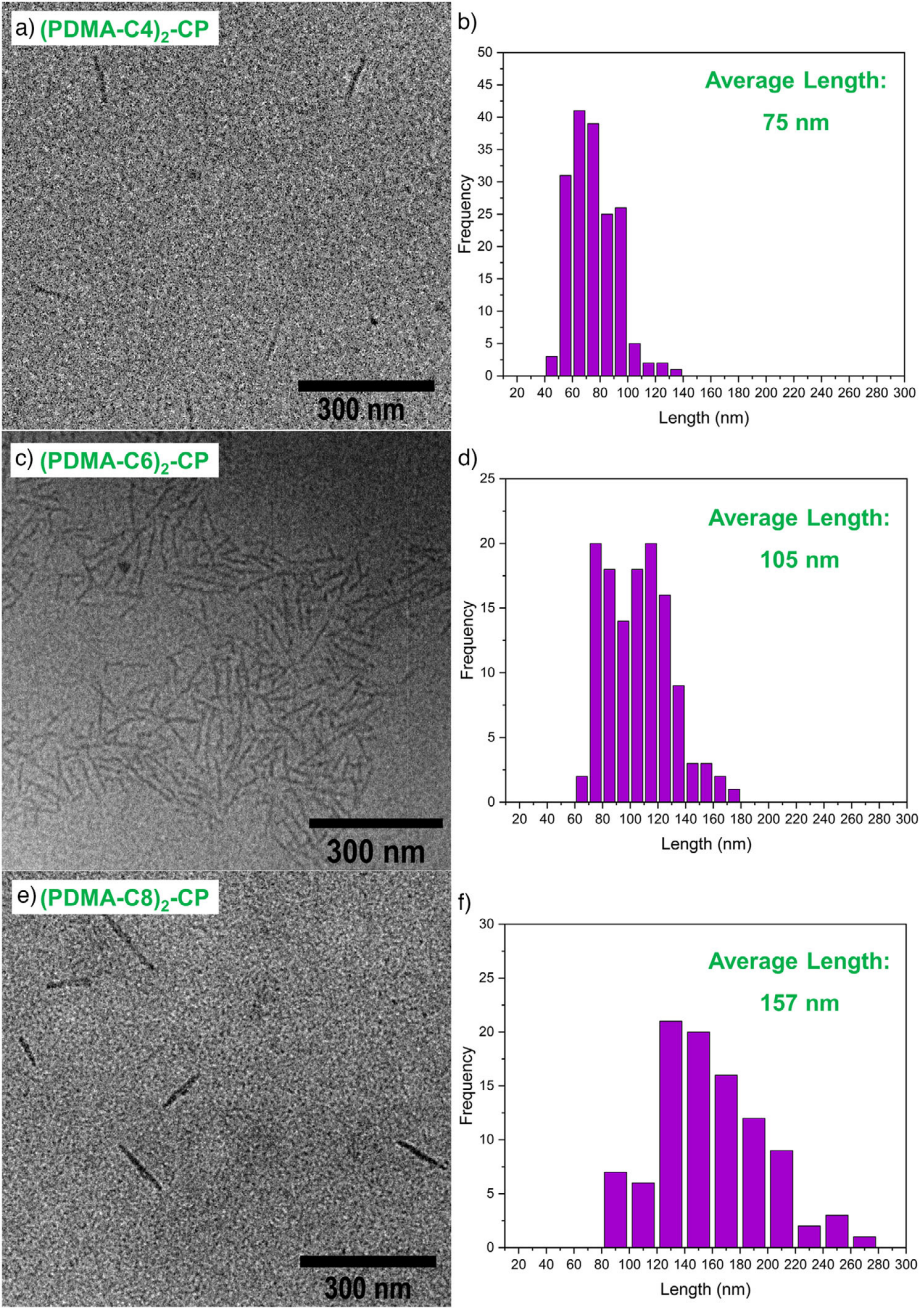
TEM images of a) (PDMA‐C4)_2_‐CP, c) (PDMA‐C6)_2_‐CP, and e) (PDMA‐C8)_2_‐CP nanotubes. The calculated average length of the nanotubes from TEM images: b) (PDMA‐C4)_2_‐CP, d) (PDMA‐C6)_2_‐CP, and f) (PDMA‐C8)_2_‐CP.

Transmission electron microscopy (TEM) was used to image the morphology of the assembled nanotubes. Elongated cylinders were observed for (PDMA‐C4)_2_‐CP, (PDMA‐C6)_2_‐CP, and (PDMA‐C8)_2_‐CP conjugates (Figure 2a,c,e) with average length of 75, 105, and 157 nm (Figure 2b,d,f), respectively. The average width of these nanotubes was less than 20 nm (Figure S17). No nanotubes were observed for (PDMA‐C2)_2_‐CP. Interestingly, the length of nanotubes increased with the length of hydrophobic alkyl linker group in the conjugate (Table S2). Considering that assembled structures were not observed in the AF_4_ analysis for the (PDMA‐C4)_2_‐CP and (PDMA‐C6)_2_‐CP conjugates, it suggests that these compounds exist as unimers in more dilute aqueous solution but nanotubes can form at higher concentrations such as during the drying process when preparing the TEM sample grids as the evaporation of water increases the concertation of the conjugates.

### Investigating Core Hydrophobic Shielding Using Molecular Dynamics Simulations

Molecular dynamics simulations were used to investigate the role of the hydrophobic linker in modulating nanotube stability. Model systems, consisting of eight unimers stacked in an antiparallel arrangement, were made by solvating prebuilt octameric nanotubes in water. Systems were modelled using the OPLS4 forcefield and Desmond.^[^ ^42^, ^43^ ^]^ Four systems were prepared that included C2 and C8 linkers capped with a short polymer (PDMA_6_) and a full‐length PDMA_50_ polymer (Table 2). The stability of the nanotube core over the course of each simulation was evaluated by counting the number of intermolecular peptide backbone hydrogen bond interactions. In a stacked arrangement, eight hydrogen bonds can be formed between each cyclic peptide interface, and as there are seven intermolecular interfaces in an assembled octamer, a maximum of 56 hydrogen bonds can form. Sixteen of the H‐bonds come from capping unimers and 40 H‐bonds from the core of the nanotube (Figure S18). The averaged distance between unimers is 4.87 and 4.85 Å for (PDMA_6_‐C2)_2_‐CP and (PDMA_6_‐C8)_2_‐CP, respectively (Figure S19).

**Table 2:** Structures of the unimers modeled using molecular dynamics simulations. In each simulation, eight unimers were assembled to form an octamer nanotube.

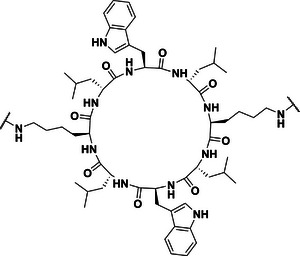			
Sim	Length (ns)	Sample	Linker–polymer unit
1	1500	(PDMA_6_‐C2)_2_‐CP	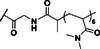
2	1500	(PDMA_6_‐C8)_2_‐CP	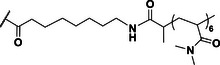
3	100	(PDMA_50_‐C2)_2_‐CP	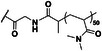
4	100	(PDMA_50_‐C8)_2_‐CP	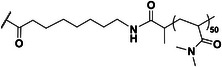

**Figure 3:**
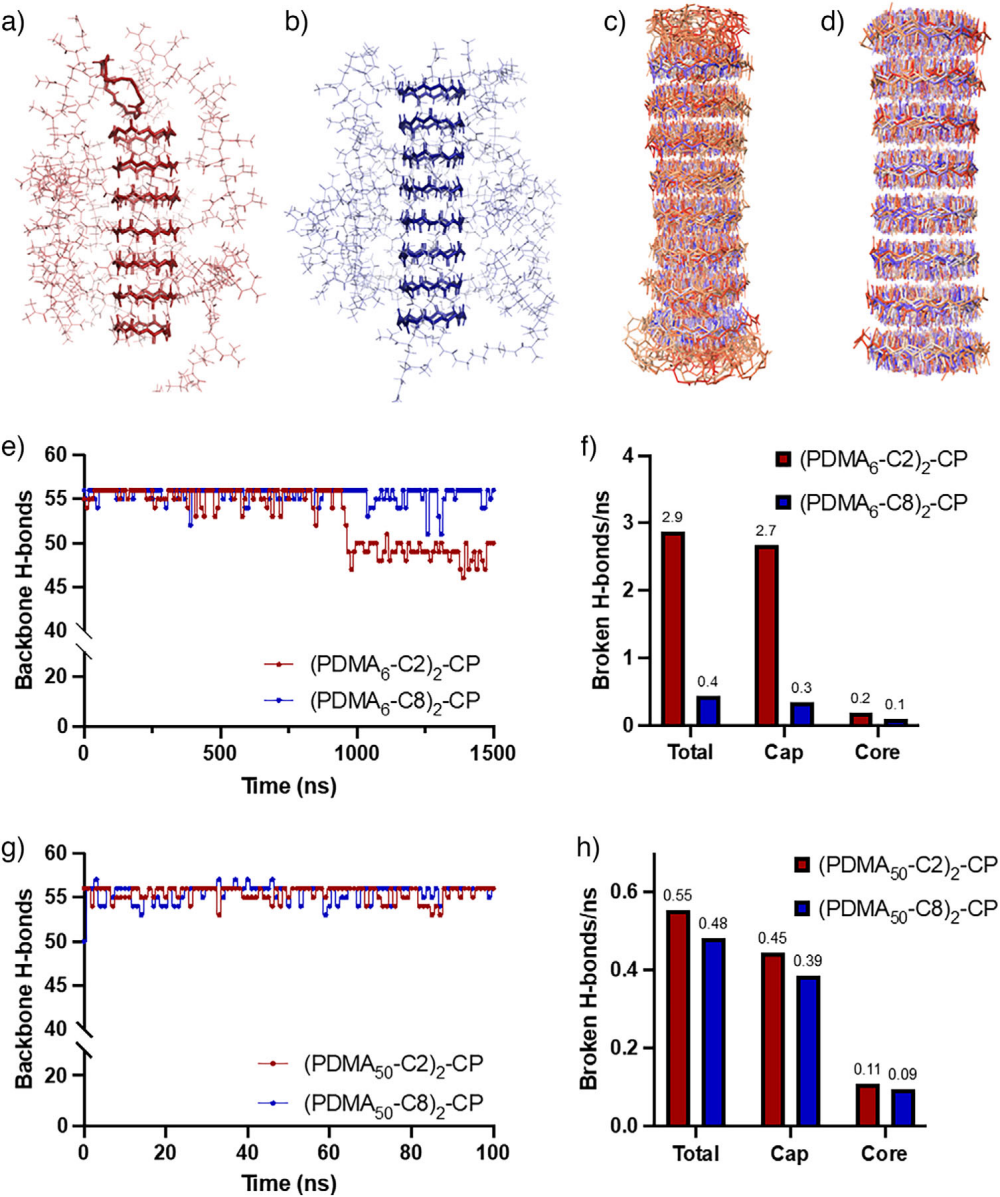
Molecular dynamics simulations of CP‐linker‐polymer nanotubes. Final frames of simulations of a) (PDMA_6_‐C2)_2_‐CP and b) (PDMA_6_‐C8)_2_‐CP. The peptide backbone is bold, polymer is shown as lines, and CP sidechains are omitted for clarity. Overlays of the CP core alone (sidechains, linker, and polymer are not shown) sampled over the course of the simulation for c) (PDMA_6_‐C2)_2_‐CP and d) (PDMA_6_‐C8)_2_‐CP. Total counts of nanotube backbone–backbone H‐bond interactions over the course of simulation for e) (PDMA_6_‐C2)_2_‐CP (red) and (PDMA_6_‐C8)_2_‐CP (blue) and g) (PDMA_50_‐C2)_2_‐CP (red) and (PDMA_50_‐C8)_2_‐CP (blue). Number of H bonds broken per nanosecond for f) (PDMA_6_‐C2)_2_‐CP (red) and (PDMA_6_‐C8)_2_‐CP (blue) and h) (PDMA_50_‐C2)_2_‐CP (red) and (PDMA_50_‐C8)_2_‐CP (blue) nanotubes comparing total, cap, and core.

The relatively small size of the systems containing a short PDMA_6_ polymer (**Sims 1**, **2**) allowed us to run simulations for 1500 ns. The final frames of these simulations are shown in Figure 3a,b. The octamer with a C8 linker was stable over the entire simulation, whereas the C2 linker nanotube was less stable and there was greater fluctuation in the capping group backbone hydrogen bonds (Figure 3c–e). At the 1000 ns time point, the backbone hydrogen bond count of the C2‐linker nanotube drops by ∼8, which corresponds to the unbinding of a capping peptide.

The nanotube backbone hydrogen bonds are dynamic, breaking and reforming throughout the course of the simulation. We used the number of broken hydrogen bonds per nanosecond (BHB ns^−1^) as a metric to measure the relative stability of the C2 and C8 linker nanotube systems and to compare the stability of cap and core peptides (Figure 3f). The C2 system, with a total of 2.9 BHB ns^−1^, was markedly less stable than the C8 system with 0.4 BHB ns^−1^. Hydrogen bonds between the capping (terminal) peptides are much less stable than the core peptides and contribute most to the total count.

During the simulation of the C2 linker mode (**Sim 1**), one capping group was observed to partially dissociate from the nanotube (Figure 3a). Examination of trajectory shows that the mechanism is initiated by a water molecule displacing backbone hydrogen bonds from the interior of the nanotube. As the backbone hydrogen bonds of the cap peptide are broken, water from the exterior of the nanotube enters and occupies the remaining hydrogen bond interactions of that interface. In comparison, the C8 system overcame these attacks from the interior pore water because more hydrophobic C8 alkyl chains anchor to the core peptides and prevent complete dissociation, stopping the attack from exterior water molecules.

This observed mechanism of dissociation is consistent with previous studies performed by our group where it was found that the nanotubes followed a cooperative assembly mechanism.^[^ ^30^ ^]^ Cooperative assembly requires a nucleation event to occur before peptide unimers add spontaneously to lengthen the nanotube. Capping peptide unimers, therefore, behave differently from the core of the structure and are the dynamic components that ultimately regulate the length of the nanotube. The molecular dynamics simulations suggest that the capping unimers with a C2 linker are much less stable than those with the C8 linker, showing that the presence of a hydrophobic linker significantly affects capping group dynamics.

To create a more complete representation of the experimental systems, we modeled systems conjugated with the full‐length hydrophilic polymer with a DP of 50 (**Sims 3 and 4**; Figures S20 and S21). Due to the large system size (∼5 70 000 atoms), the models could only be run for 100 ns. An animation of the simulation is provided as Supporting Information. The simulation started with the polymer chains in an extended conformation that then quickly collapsed, intertwining with one another and encasing the nanotube core. This observation came as a surprise as our work to date had suggested the polymeric chains extend in solution to provide steric stabilization. The simulation shows that, instead, the chains collapse and wrap around the peptide nanotube, providing additional stability to the assemblies. The core backbone hydrogen bond count shows both C2 and C8 nanotubes were stable over the course of the simulation (Figure 3g). Comparing the capping peptide unimer hydrogen bonds, both the C2 and C8 system have small differences in broken hydrogen bonds per nanosecond, 0.45 and 0.39 BHB ns^−1^, respectively (Figure 3h). The C8 system was more stable than C2 system in 100 ns simulation, and we suggest that longer simulations will be able to observe more significant differences.

Taken together, the molecular dynamics simulations and experimental measurements are consistent. Incorporation of a hydrophobic region between the peptide and polymer increases stability and that the stability increase depends on the size of the hydrophobic linker. The simulations show that the linker makes a hydrophobic inner shell that prevents water from attacking and breaking the peptide backbone hydrogen bonds. The linker has the greatest effect on the unimers that cap the nanotube, which have the least stable backbone hydrogen bonding. Therefore, modulating the stability of the capping unimers by incorporating a defined, short hydrocarbon linker provides an avenue to tune the size and dynamic nature of the resulting self‐assembled nanostructures.

## Conclusion

In this work, we have integrated experimental and theoretical approaches to develop methods for controlling the self‐assembly of CP nanotubes in water. A series of cyclic peptide–polymer conjugates with varied length of hydrophobic alkyl linker group were synthesized and assembled into nanotubes. The interplay among hydrophilic interactions of polymer chain, hydrophobic interactions of the alkyl group, and H‐bonds of cyclic peptide resulted in the formation of nanotubes in water. The highly dynamic self‐assembling behavior of these conjugates was observed, and the length of the nanotubes was regulated by the length of hydrophobic alkyl linker group. Molecular dynamics simulations elucidated the mechanism of assembly and demonstrated that the more hydrophobic linker results in more stable nanotubes. The hydrophobic domain adjacent to the CP core protects the hydrogen‐bonded backbone from water, and longer hydrophobic linker increases the stability of the more labile capping unimers. In addition, the simulations show how, unexpectedly, the polymeric chains wrap around the peptide nanotubes, which provides additional stability to the assemblies, rather than extending in solution as expected. This work improves our understanding of CP nanotubes assembly and stability and provides a facile route for the design of more advanced CP nanotube materials.

## Supporting Information

Synthetic protocols and characterization of all materials, fitting parameters of SANS data, structures obtained from molecular dynamics simulations. The authors have cited additional references within the Supporting Information.^[^ ^5^, ^43^, ^44^, ^45^, ^46^, ^47^ ^]^

## Conflict of Interests

The authors declare no conflict of interest.

## Supporting information



Supporting Information

Supporting Information

## Data Availability

The data that support the findings of this study are available from the corresponding author upon reasonable request.

## References

[1] M. R. Ghadiri , J. R. Granja , R. A. Milligan , D. E. McRee , N. Khazanovich , Nature 1993, 366, 324–327. 8247126 10.1038/366324a0

[2] R. Hourani , C. Zhang , R. van der Weegen , L. Ruiz , C. Li , S. Keten , B. A. Helms , T. Xu , J. Am. Chem. Soc. 2011, 133, 15296–15299. 21894889 10.1021/ja2063082

[3] R. Chapman , M. Danial , M. L. Koh , K. A. Jolliffe , S. Perrier , Chem. Soc. Rev. 2012, 41, 6023. 22875035 10.1039/c2cs35172b

[4] D. J. Rubin , S. Amini , F. Zhou , H. Su , A. Miserez , N. S. Joshi , ACS Nano 2015, 9, 3360–3368. 25757883 10.1021/acsnano.5b00672

[5] M. R. Silk , J. Newman , J. C. Ratcliffe , J. F. White , T. Caradoc‐Davies , J. R. Price , S. Perrier , P. E. Thompson , D. K. Chalmers , Chem. Commun. 2017, 53, 6613–6616. 10.1039/c7cc00846e 28581562

[6] M. R. Silk , J. R. Price , B. Mohanty , H.‐K. S. Leiros , B. A. Lund , P. E. Thompson , D. K. Chalmers , Chem. Eur. J. 2021, 27, 14489–14500. 34415083 10.1002/chem.202102106

[7] S. Goswami , A. Reja , S. Pal , A. Singh , D. Das , J. Am. Chem. Soc. 2022, 144, 19248–19252. 36219699 10.1021/jacs.2c09262

[8] M. Kobayashi , K. Fujita , K. Matsuda , T. Wakimoto , J. Am. Chem. Soc. 2023, 145, 3270–3275. 36638272 10.1021/jacs.2c11082

[9] J. Miao , M. L. Descoteaux , Y.‐S. Lin , Chem. Sci. 2021, 12, 14927–14936. 34820109 10.1039/d1sc05562c PMC8597836

[10] L. Yu , S. A. Barros , C. Sun , S. Somani , J. Chem. Inf. Model. 2023, 63, 6863–6876. 37903231 10.1021/acs.jcim.3c01359

[11] R. Vijayaraj , S. Van Damme , P. Bultinck , V. Subramanian , Phys. Chem. Chem. Phys. 2012, 14, 15135. 23041975 10.1039/c2cp42030a

[12] R. Moral , S. Paul , Phys. Chem. Chem. Phys. 2023, 25, 5406–5422. 36723368 10.1039/d2cp05160e

[13] J. Montenegro , M. R. Ghadiri , J. R. Granja , Acc. Chem. Res. 2013, 46, 2955–2965. 23898935 10.1021/ar400061d PMC3867521

[14] M. Danial , C. M. N. Tran , K. A. Jolliffe , S. Perrier , J. Am. Chem. Soc. 2014, 136, 8018–8026. 24810461 10.1021/ja5024699

[15] A. Kerr , E. Sagita , E. D. H. Mansfield , T.‐H. Nguyen , O. M. Feeney , C. W. Pouton , C. J. H. Porter , J. Sanchis , S. Perrier , Biomacromolecules 2022, 23, 2315–2328. 35582852 10.1021/acs.biomac.2c00063 PMC9198979

[16] A. Lamas , A. Guerra , M. Amorín , J. R. Granja , Chem. Sci. 2018, 9, 8228–8233. 30542571 10.1039/c8sc02276c PMC6240800

[17] S. C. Larnaudie , J. Sanchis , T.‐H. Nguyen , R. Peltier , S. Catrouillet , J. C. Brendel , C. J. H. Porter , K. A. Jolliffe , S. Perrier , Biomaterials 2018, 178, 570–582. 29680158 10.1016/j.biomaterials.2018.03.047

[18] A. Méndez‐Ardoy , J. R. Granja , J. Montenegro , Nanoscale Horiz. 2018, 3, 391–396. 32254126 10.1039/c8nh00009c

[19] A. Bayón‐Fernández , A. Méndez‐Ardoy , C. Alvarez‐Lorenzo , J. R. Granja , J. Montenegro , J. Mater. Chem. B 2023, 11, 606–617. 36533555 10.1039/d2tb01721k

[20] S. Fernandez‐Lopez , H.‐S. Kim , E. C. Choi , M. Delgado , J. R. Granja , A. Khasanov , K. Kraehenbuehl , G. Long , D. A. Weinberger , K. M. Wilcoxen , M. R. Ghadiri , Nature 2001, 412, 452–455. 11473322 10.1038/35086601

[21] M. Hartlieb , S. Catrouillet , A. Kuroki , C. Sanchez‐Cano , R. Peltier , S. Perrier , Chem. Sci. 2019, 10, 5476–5483. 31293730 10.1039/c9sc00756c PMC6544120

[22] Q. Song , S. Goia , J. Yang , S. C. L. Hall , M. Staniforth , V. G. Stavros , S. Perrier , J. Am. Chem. Soc. 2021, 143, 382–389. 33348987 10.1021/jacs.0c11060 PMC8172009

[23] H. Lu , Y. Wang , S. K. Hill , H. Jiang , Y. Ke , S. Huang , D. Zheng , S. Perrier , Q. Song , Angew. Chem. Int. Ed. 2023, 62, e202311224. 10.1002/anie.202311224 37840434

[24] M. R. Silk , B. Mohanty , J. B. Sampson , M. J. Scanlon , P. E. Thompson , D. K. Chalmers , Angew. Chem. Int. Ed. 2019, 58, 596–601. 10.1002/anie.201811910 30452108

[25] L. Ayres , G. M. Grotenbreg , G. A. van der Marel , H. S. Overkleeft , M. Overhand , J. C. M. van Hest , Macromol. Rapid Commun. 2005, 26, 1336–1340.

[26] M. G. J. ten Cate , N. Severin , H. G. Börner , Macromolecules 2006, 39, 7831–7838.

[27] J. Couet , M. Biesalski , Soft Matter 2006, 2, 1005. 32680203 10.1039/b611846c

[28] R. Chapman , G. G. Warr , S. Perrier , K. A. Jolliffe , Chem. Eur. J. 2013, 19, 1955–1961. 23297172 10.1002/chem.201203602

[29] R. Chapman , K. A. Jolliffe , S. Perrier , Adv. Mater. 2013, 25, 1170–1172. 23288610 10.1002/adma.201204094

[30] R. Chapman , M. L. Koh , G. G. Warr , K. A. Jolliffe , S. Perrier , Chem. Sci. 2013, 4, 2581.

[31] J. Y. Rho , H. Cox , E. D. H. Mansfield , S. H. Ellacott , R. Peltier , J. C. Brendel , M. Hartlieb , T. A. Waigh , S. Perrier , Nat. Commun. 2019, 10, 4708. 31624265 10.1038/s41467-019-12586-8 PMC6797743

[32] J. Y. Rho , S. Perrier , ACS Macro Lett. 2021, 10, 258–271. 35570781 10.1021/acsmacrolett.0c00734

[33] Q. Song , Z. Cheng , M. Kariuki , S. C. L. Hall , S. K. Hill , J. Y. Rho , S. Perrier , Chem. Rev. 2021, 121, 13936–13995. 33938738 10.1021/acs.chemrev.0c01291 PMC8824434

[34] J. Couet , J. D. J. S. Samuel , A. Kopyshev , S. Santer , M. Biesalski , Angew. Chem. Int. Ed. 2005, 44, 3297–3301. 10.1002/anie.200462993 15830332

[35] J. Y. Rho , J. C. Brendel , L. R. MacFarlane , E. D. H. Mansfield , R. Peltier , S. Rogers , M. Hartlieb , S. Perrier , Adv. Funct. Mater. 2018, 28, 1704569.

[36] J. C. Brendel , J. Sanchis , S. Catrouillet , E. Czuba , M. Z. Chen , B. M. Long , C. Nowell , A. Johnston , K. A. Jolliffe , S. Perrier , Angew. Chem. Int. Ed. 2018, 57, 16678–16682. 10.1002/anie.201808543 30383920

[37] J. Yang , J.‐I. Song , Q. Song , J. Y. Rho , E. D. H. Mansfield , S. C. L. Hall , M. Sambrook , F. Huang , S. Perrier , Angew. Chem. Int. Ed. 2020, 59, 8860–8863. 10.1002/anie.201916111 32045099

[38] J. Yang , X. Yu , J.‐I. Song , Q. Song , S. C. L. Hall , G. Yu , S. Perrier , Angew. Chem. Int. Ed. 2022, 61, e202115208. 10.1002/anie.202115208 34927320

[39] J. R. Runyon , M. Ulmius , L. Nilsson , Colloids Surf. A Physicochem. Eng. Asp. 2014, 442, 25–33.

[40] H. Zhang , D. Lyden , Nat. Protoc. 2019, 14, 1027–1053. 30833697 10.1038/s41596-019-0126-x PMC6733524

[41] M. Kariuki , J. Y. Rho , S. C. L. Hall , S. Perrier , Macromolecules 2023, 56, 6618–6632. 37720562 10.1021/acs.macromol.3c00442 PMC10501196

[42] K. J. Bowers , D. E. Chow , H. Xu , R. O. Dror , M. P. Eastwood , B. A. Gregersen , J. L. Klepeis , I. Kolossvary , M. A. Moraes , F. D. Sacerdoti , J. K. Salmon , Y. Shan , D. E. Shaw , in Proceed. ACM/IEEE SC2006 Conf. High Perf. Network. Comp., ACM Press, Tampa 2006, pp. 43–43.

[43] C. Lu , C. Wu , D. Ghoreishi , W. Chen , L. Wang , W. Damm , G. A. Ross , M. K. Dahlgren , E. Russell , C. D. Von Bargen , R. Abel , R. A. Friesner , E. D. Harder , J. Chem. Theory Comput. 2021, 17, 4291–4300. 34096718 10.1021/acs.jctc.1c00302

[44] S. A. Raw , Tetrahedron Lett. 2009, 50, 946–948.

[45] G. J. Martyna , M. L. Klein , M. Tuckerman , J. Chem. Phys. 1992, 97, 2635–2643.

[46] G. J. Martyna , D. J. Tobias , M. L. Klein , J. Chem. Phys. 1994, 101, 4177–4189.

[47] T. Darden , D. York , L. Pedersen , J. Chem. Phys. 1993, 98, 10089–10092.

