# Supplementary material for: Hydrophobicity‐Controlled Self‐Assembly of Supramolecular Peptide Nanotubes in Water
Source: Angew Chem Int Ed Engl. 2025 Apr 14;64(22):e202423828. doi: 10.1002/anie.202423828 (PMC12105707; doi:10.1002/anie.202423828)
Supplement: Supplementary file 1 — Supporting Information [file ANIE-64-e202423828-s001.pdf]

Supporting Information

Hydrophobicity-Controlled Self-Assembly of Supramolecular Peptide Nanotubes in Water

Min Zeng,§[a] William Parsons,§[b] Yixuan Chen,[a] David K. Chalmers,*[b] and Sébastien Perrier*[a][b][c]

[a]Department of Chemistry, University of Warwick, Coventry CV4 7AL, United Kingdom

[b]Monash Institute of Pharmaceutical Sciences, Monash University, Parkville, Victoria 3052, Australia

[c]Warwick Medical School, University of Warwick, Coventry, CV4 7AL, United Kingdom

*Corresponding Authors

E-mail address: david.chalmers@monash.edu (David K. Chalmers)

E-mail address: s.perrier@warwick.ac.uk (Sébastien Perrier)

§These authors contributed equally.

Experimental Section

1.1 Materials.

N,*N*-Dimethylacrylamide (DMA, Sigma-Aldrich, 99%) was purified by passing through a neutral alumina oxide column to remove inhibitor before polymerization. 2,2′-azobis(2-methylpropionitrile) (AIBN, Sigma-Aldrich, 98%) was recrystallized in ethanol before use. 4-(4,6-Dimethoxy-1,3,5-triazin-2-yl)-4-methylmorpholinium tetrafluoroborate (DMTMM·BF_4) was synthesized according to literature protocol.[1] Fmoc-D-Leu-OH, Fmoc-L-Trp(Boc)-OH and Fmoc-L-Lys(Boc)-OH were bought from Iris Biotech GmbH and used as received. 1-Butanethiol (99%), carbon disulfide (≥ 99.9%), 2-bromopropionic acid (99%), *N*-hydroxysuccinimide (NHS, 98%), 4-dimethylaminopyridine (DMAP, ≥ 99%), *N*-(3-dimethylaminopropyl)-*N*′-ethylcarbodiimide hydrochloride (EDCI), glycine (C2, ≥ 99%), 4-aminobutyric acid (C4, ≥ 99%), 6-aminocaproic acid (C6, ≥ 99%), 8-aminocaprylic acid (C8, 99%), piperidine (99%), *N*,*N*-diisopropylethylamine (DIPEA, ≥ 99%), 2-(6-chloro-1-H-benzotriazole-1-yl)-1,1,3,3-tetramethylaminium hexafluorophosphate (HCTU), 1-[bis(dimethylamino)methylene]-1H-1,2,3-triazolo[4,5-b]pyridinium 3-oxid hexafluorophosphate (HATU, ≥ 98%) 4-methylmorpholine (NMM, 99%), triisopropylsilane (TIPS, 98%), 2-chlorotrityl chloride resin (100-200 mesh, 1% DVB) were purchased from Sigma-Aldrich. 1,1,1,3,3,3-Hexafluoro-2-propanol (HFIP, 99.5%), trifluoroacetic acid (TFA, 99%), sodium hydroxide (NaOH) pellets, hydrochloric acid (HCl, 37%), anhydrous magnesium sulfate ($MgSO_4$) and alumina oxide were purchased from Fisher. All solvents were purchased from several suppliers, including Sigma-Aldrich, Fisher and Honeywell.

1.2 Instrumentations.

Proton nuclear magnetic resonance (^1H NMR) spectra were recorded on a Bruker Avance III HD 400 MHz spectrometer at 25 °C, using $CDCl_3$, D_2O or TFA-*d* as solvent.

Electrospray ionization mass spectrometry (ESI-MS) measurements were performed on an Agilent 6120B single Quad.

The size exclusion chromatography (SEC) analyses were conducted on an Agilent Infinity II MDS instrument at 50 °C with a DMF with 0.1% LiBr flow rate of 1 mL min^{-1}. The DMF SEC system was

equipped with differential refractive index (DRI), variable wavelength UV detectors, viscometry (VS), and dual angle light scatter (LS) detectors. The system was equipped with 2 x PolarGel-M Columns (300 x 7.5 mm) and a PolarGel 5 μm guard column. The apparent molecular weights were calculated based on a series of poly(methyl methacrylate) standards with molecular weight range of 550-955000 g mol^{-1}.

Transmission electron microscopy (TEM) images were recorded on a 200 kV JEOL 2100 PLUS transmission electron microscope. For the preparation of TEM samples, 10 μL of 1 mg mL^{-1} sample solution was dropped onto a piece of copper grid, and the droplet was removed by filter paper after 60 s. This process was repeated for three times. The grid was further stained by the vapor from a solution of 0.5 wt% ruthenium tetroxide (RuO_4) in water. The copper grid was dried at room temperature before TEM observation.

Asymmetric flow field flow fractionation (AF_4) analyses were performed on the PostNova Analytics AF2000 multiflow system. The system was equipped with differential refractive index (RI) PN3150 detector, multiangle light scattering (MALS) detector, and single wavelength UV-vis detector. The sample separation was occurred inside a channel with a 350 μm thickness polytetrafluoroethylene (PTFE) spacer. Bovine serum albumin (BSA, 66 kDa) was used to calibrate the RI and MALS detectors, and polystyrenesulfonate sodium salt solutions (PSS, 63.9 kDa) was used to normalize the scattering angles of MALS detector. Analyte samples were prepared in water with the concentration of 1 mg mL^{-1}.

Small angle neutron scattering (SANS) was measured on Larmor at the ISIS Pulsed Neutron Source (STFC Rutherford Appleton Laboratory, Didcot, UK). Before the measurement, the sample was dissolved in D_2O/DMSO-d (95/5, v) to a concentration of 2 mg mL^{-1}, and placed in a 2 mm path length quartz cuvette. The scattering cross-section was measured over a Q-range of 0.004-0.5 $Å^{-1}$, where Q is defined as:

$$Q = \frac{2\pi Sin\theta}{\lambda}$$

Here, 2θ is the scattered angle, and λ is the incident neutron wavelength. A Q-range of 0.004-0.5 $Å^{-1}$ was achieved using an incident wavelength range of 0.9-13.3 Å. The detector is located 4.1 m from the sample and is 664 mm wide × 664 mm high with the beam in the centre of the detector. The beam size is 6 mm wide and 08 mm high. The raw scattering data set was corrected for the detector efficiencies, sample transmission and background scattering and converted to scattering cross-section data ($\partial\Sigma/\partial\Sigma$ *vs* Q) using the Mantid software. The data were placed on an absolute scale (cm^{-1}) using the scattering from a standard sample (a solid blend of hydrogenous and perdeuterated polystyrene) in accordance with established procedures.

1.3 Synthesis of (propanoic acid)yl butyl trithiocarbonate (PABTC, Scheme S1).

A 50 wt% NaOH (9.68 g, 0.24 mol) aqueous solution was slowly added to a mixture of butanethiol (20 g, 0.22 mol) and acetone (11 mL) in a 500 mL round bottom flask. Water (40 mL) was then added, the solution was stirred for 30 min at room temperature. Carbon disulfide (17.32 g, 0.23 mol) was added to the solution and stirred for 30 min. Cooling the solution to < 10 °C in ice bath, 2-bromopropionic acid (34.9 g, 0.23 mol) was slowly added, followed by the addition of 50 wt% NaOH (9.68 g, 0.24 mol) aqueous solution. The resulting solution was stirred for overnight at room temperature. Water (200 mL) was added to the reaction mixture. HCl solution (10 M) was dropwise added to the solution, the orange solid was precipitated. The crude product was separated and recrystallised in hexane to obtain the orange PABTC (36.00 g) crystal. Yield: 69%. ^{1}H NMR ($CDCl_3$, 400 MHz, pm): δ = 4.86 (q, 1H, J = 9 Hz, C*H*(CH_3)), 3.38 (t, 2H, J = 9 Hz, S-CH_2-CH_2), 1.68 (m, 2H, S-CH_2-CH_2-CH_2), 1.62 (d, 3H, J = 9 Hz, CH(CH_3)), 1.43 (m, 2H, CH_2-CH_2-CH_3), 0.94 (t, 3H, J = 9 Hz, CH_2-CH_3) (**Figure S1**).

Scheme S1. Synthetic route of PABTC.

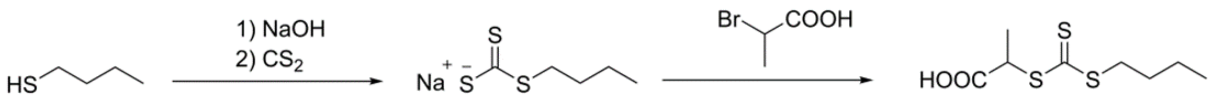

1.4 Synthesis of PABTC-NHS (Scheme S2).

PABTC (5.00 g, 20.97 mmol), NHS (2.90 g, 25.20 mmol), DMAP (256.20 mg, 2.10 mmol) were dissolved into 100 mL dichloromethane (DCM) in a 250 mL round bottom flask. A solution of EDCI

(4.83 g, 25.20 mmol, dissolved in 80 mL DCM) was dropwise added into the flask. The mixture solution was stirred for overnight. The DCM solution was washed with water (3 x 50 mL) and brine (3 x 50 mL). The collected organic layer was dried with anhydrous $MgSO_4$. The DCM solution was concentrated by rotary evaporation and purified via silica gel chromatography (hexane/ethyl acetate = 7/3, v) to obtain PABTC-NHS (5.80 g, 17.29 mmol) as a yellow oil. Yield: 82%. ^{1}H NMR ($CDCl_3$, 400 MHz, pm): δ = 5.15 (q, 1H, J = 9 Hz, C*H*(CH_3)), 3.38 (t, 2H, J = 9 Hz, S-CH_2-CH_2), 2.83 (s, 4H, succinimidyl CH_2-CH_2), 1.71 (d, 3H, J = 9 Hz, CH(CH_3)), 1.70 (m, 2H, S-CH_2-CH_2-CH_2), 1.44 (m, 2H, CH_2-CH_2-CH_3), 0.93 (t, 3H, J = 9 Hz, CH_2-CH_3) (**Figure S2**).

Scheme S2. Synthetic route of PABTC-NHS.

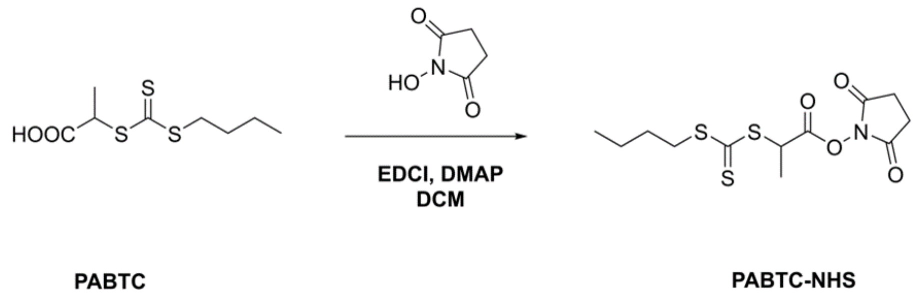

1.5 Synthesis of PDMA-NHS (Scheme S3).

PABTC-NHS (651.1 mg, 1.94 mmol), DMA (9.62 g, 97.04 mmol), and AIBN (63.7 mg, 0.39 mmol) were weighed into a Schlenk flask, then 1,4-dioxane (30 mL) was added. The sealed flask was degassed with N_2 for 30 min to remove oxygen before being placed into a preheated oil bath at 70 °C. After 2 h, the polymerization was quenched in ice bath, and the monomer conversion (99%) was characterized by ^{1}H NMR analysis. Then the reaction solution was precipitated into diethyl ether, and dried under vacuum to obtain PDMA-NHS as a yellow solid powder. DMF SEC was used to characterize the molecular weight ($M_{n,SEC}$ = 4700) and dispersity ($Đ$ = 1.18).

Scheme S3. Synthetic route of PDMA-NHS.

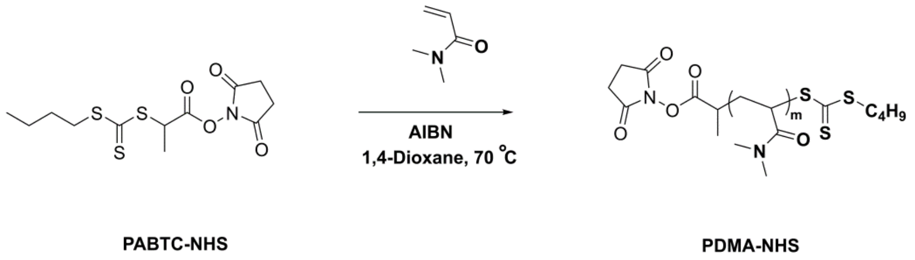

1.6 Synthesis of PDMA-Linker (Scheme S4).

The hydrophilic polymer PDMA-NHS was modified by a series of small molecules with varied length of alkyl group. In a typical protocol, PDMA-NHS (1.00 g, 0.21 mmol), 6-aminocaproic acid (31.5 mg, 0.24 mmol) and EDCI (38.2 mg, 0.20 mmol) were dissolved in 5 mL water. The solution was stirred for overnight and then dialysis against with water. The aqueous solution was dried by freeze dryer to afford the PDMA-C6 as a light-yellow powder.

Using the same protocol, PDMA-C2, PDMA-C4 and PDMA-C8 were synthesized.

Scheme S4. Synthetic route of PDMA-C_x (x = 2, 4, 6, and 8).

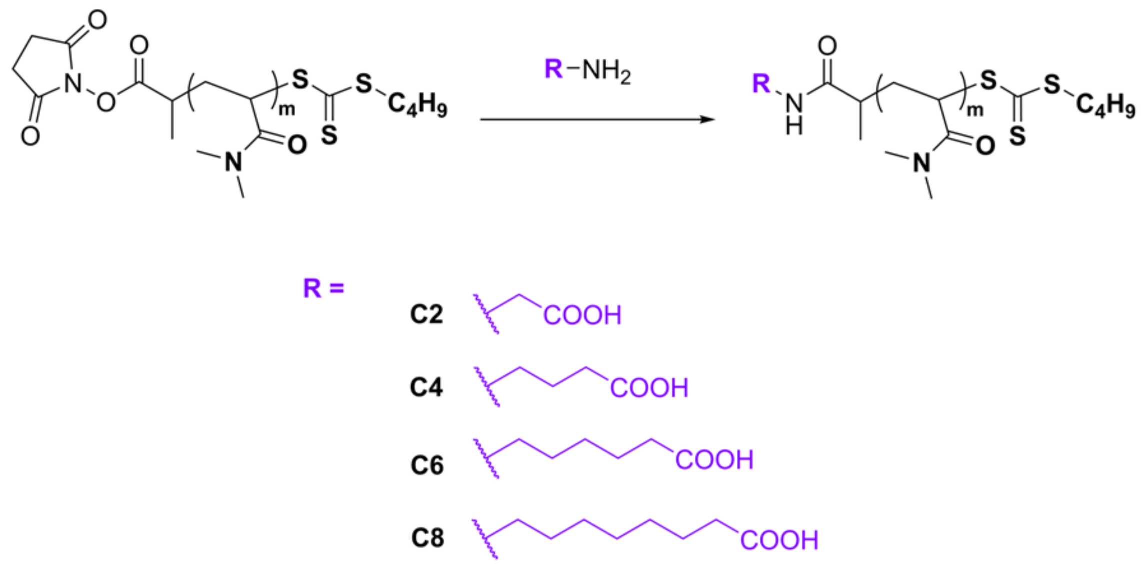

1.7 Synthesis of Linear Peptide (LP, Scheme S5).

The linear peptide H_2N-L-Lys(Boc)-D-Leu-L-Trp(Boc)-D-Leu-L-Lys(Boc)-D-Leu-L-Trp(Boc)-D-Leu-COOH was prepared via solid phase peptide synthesis (SPPS) method. The synthesis was performed

on a Prelude Peptide Synthesizer (Protein Technologies, Inc.), using 2-chlorotrityl chloride resin as the solid support. The resin was rinsed with DCM, and then the first amnio acid was loaded onto the resin using DIPEA as coupling agent. The unreacted resin sites were capped with a mixture solution of DCM: methanol: DIPEA (17: 2: 1, v). The Fmoc protection group of amino acid was removed by using 20 % piperidine in DMF. The attachment of subsequent amino acids was using HCTU as coupling agent and NMM as base. After the entire sequence was completed, the linear peptide was cleaved from the resin using a solution of 20% HFIP in DCM. The resulting solution was concentrated by rotary evaporation and dried in vacuum to yield the linear peptide as an off-white solid. ^{1}H NMR (TFA-*d*, 400 MHz, ppm): δ = 8.07 (m, 2H, Trp), 7.54-7.22 (m, 8H, Trp), 5.11 (m, 2H, H_α Trp), 4.68-4.48 (m, 5H, H_α Leu and H_α Lys), 4.21 (m, 1H, H_α Lys N-end), 3.32-3.03 (m, 8H, CH_2 Trp and CH_2-NH Lys), 2.07-0.86 (m, 60H, CH_2-CH_2-CH_2 Lys, CH_2-CH Leu, $C(CH_3)_3$ Boc), 0.85-0.58 (m, 24H, CH_3 Leu) (**Figure S3**). MS (ESI): calculated MW: 1499.9, found $[M-H]^-$: 1498.9.

Scheme S5. Synthetic route of cyclic peptide: H_2N-CP-NH_2.

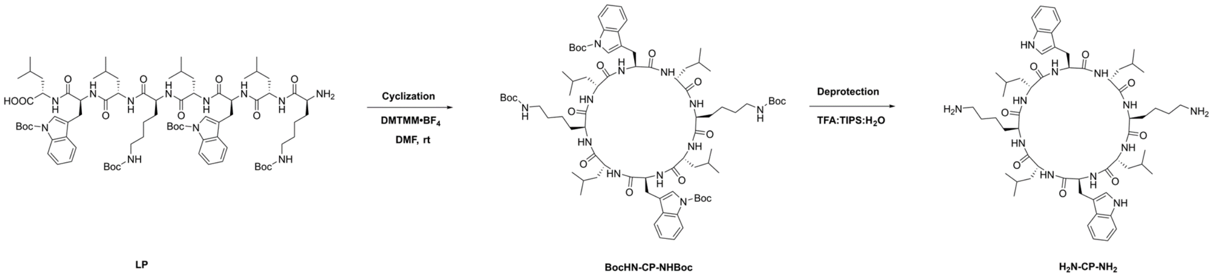

1.8 Synthesis of Protected Cyclic Peptide (BocHN-CP-NHBoc, Scheme S5).

Linear peptide (0.34 g, 0.23 mmol) was dissolved in 300 mL DMF to form a dilute solution. The coupling agent DMTMM·BF_4 (89.30 mg, 0.27 mmol) was added into the solution, followed by stirring for 48 hrs. The resulting solution was concentrated to 20 mL by rotary evaporation. The concentrated solution was precipitated into a cold mixture solvent of methanol: water (1: 1, v). The collected precipitates were dried in vacuum to obtain the protected cyclic peptide as an off-white solid. ^{1}H NMR (TFA-*d*, 400 MHz, ppm): δ = 8.07 (m, 2H, Trp), 7.54-7.22 (m, 8H, Trp), 5.15 (m, 2H, H_α Trp), 4.79-4.52 (m, 6H, H_α Leu and H_α Lys), 3.29-2.96 (m, 8H, CH_2 Trp and CH_2-NH Lys), 2.07-0.86 (m, 60H,

CH_2-CH_2-CH_2 Lys, CH_2-CH Leu, $C(CH_3)_3$ Boc), 0.85-0.58 (m, 24H, CH_3 Leu) (**Figure S4**). MS (ESI): calculated MW: 1481.9, found $[M-H]^-$: 1480.9.

1.9 Synthesis of Deprotected Cyclic Peptide (H_2N-CP-NH_2, Scheme S5).

The protected cyclic peptide (128.0 mg, 86.4 μmol) was dissolved in 2 mL mixture solution of TFA: TIPS: H_2O (18: 1: 1, v) to remove the Boc groups. The mixture was stirred for 3 hrs, and then precipitated into cold diethyl ether. The collected precipitates were dried in vacuum to obtain the deprotected cyclic peptide as an off-white solid. ^{1}H NMR (TFA-*d*, 400 MHz, ppm): δ = 7.64-6.60 (m, 10H, Trp), 5.16 (m, 2H, Hα Trp), 4.73 (m, 6H, H_α Leu and H_α Lys), 3.29-2.96 (m, 8H, CH_2 Trp and CH_2-NH Lys), 2.07-0.86 (m, 24H, CH_2-CH_2-CH_2 Lys, CH_2-CH Leu,), 0.85-0.58 (m, 24H, CH_3 Leu) (**Figure S5**). MS (ESI): calculated MW: 1081.4, found $[M+H]^+$: 1082.6.

1.10 Synthesis of Cyclic Peptide-Polymer Conjugate (Scheme S6).

The cyclic peptide-polymer conjugates were synthesis *via* grafting to method. In a typical protocol, PDMA-C6 (138.7 mg, 28.3 μmol), cyclic peptide (10.0 mg, 9.30 μmol), HATU (10.5 mg, 27.6 μmol), and DIPEA (10 μL) were added into 2 mL DMF. The resulting solution was stirred for 48 hrs and then precipitated into diethyl ether. The collected precipitates were purified by using centrifuge dialysis tube (molecular weight cut-off: 10 kDa) to obtain two-arm cyclic peptide-polymer conjugates, (PDMA-C6)$_2$-CP.

The (PDMA-C2)$_2$-CP, (PDMA-C4)$_2$-CP and (PDMA-C8)$_2$-CP conjugates were synthesized by using the same protocol.

Scheme S6. Synthetic route of cyclic peptide-polymer conjugate, (PDMA-C6)$_2$-CP.

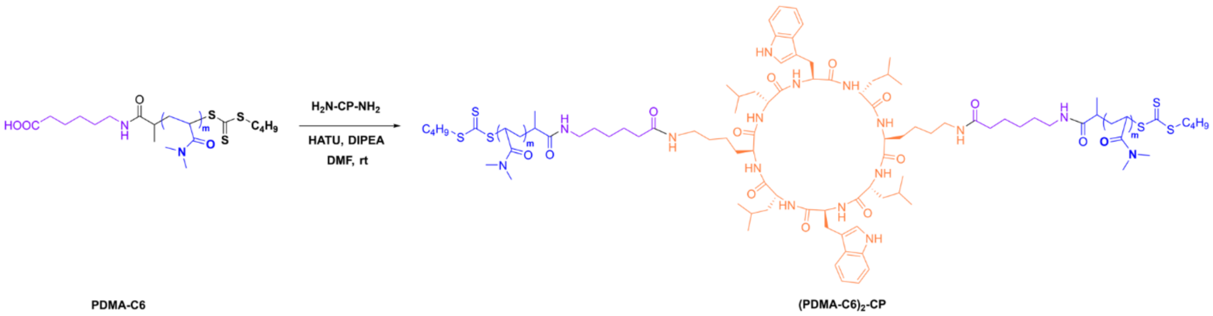

1.11 Synthesis of $PDMA_{100}$ (Scheme S7).

PABTC (23.1 mg, 97.0 μmol), DMA (962 mg, 9.70 mmol), and AIBN (2.4 mg, 14.6 μmol) were weighed into a Schlenk flask, then 1,4-dioxane (3 mL) was added. The sealed flask was degassed with N_2 for 15 min to remove oxygen before being placed into a preheated oil bath at 70 °C. After 2 h, the polymerization was quenched in ice bath, and the monomer conversion (99%) was characterized by ^{1}H NMR analysis. Then the reaction solution was precipitated into diethyl ether, and dried under vacuum to obtain PDMA as a yellow solid powder. DMF SEC was used to characterize the molecular weight ($M_{n,SEC}$ = 10500) and dispersity ($Đ$ = 1.17).

Scheme S7. Synthetic route of $PDMA_{100}$.

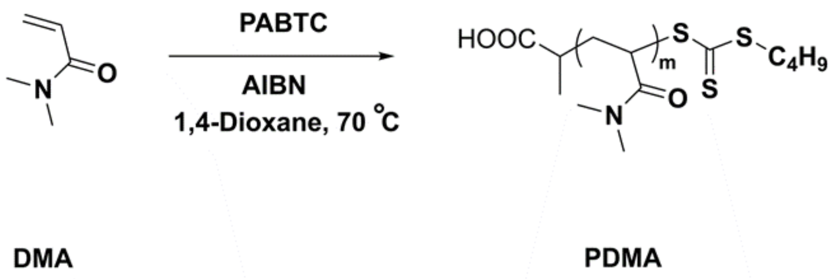

1.12 Molecular Dynamics Simulation.

Model systems were built in Maestro (Schrodinger version 13.1.141 Release 22.1) and simulated using the integrated Desmond MD software (D. E. Shaw Research version 6.9). All simulations used the OPLS4 force field.[2] Cyclic peptides were built using backbone coordinates from in house structures and residues were switched using the Maestro Build function.[3] Linker and polymer functionality was added to the peptide manually using the Maestro draw function. Octamer models were created by replicating eight peptide/polymer unimer structures in an antiparallel arrangement. Each octamer system was minimised in implicit water for 2500 iterations with constraints on backbone hydrogen bond interactions. Systems were placed in an orthorhombic cell with a buffered area of 20 x 20 x 20 Å and solvated in TIP3P water. The prepared systems were simulated using an NPT ensemble at 300 K and 1.01325 bar with a 2 fs time step. A Nose-Hoover chain method was employed for the thermostat[4] and a Martyna-Tobias-Klein method for the barostat method.[5] Simulations used periodic boundary

conditions and the particle-mesh Ewald method[6] for long range electrostatic interactions with a cutoff radius of 9.0 Å. The stability of the assembled nanotube was evaluated by the number of peptide backbone hydrogen bond interactions in the recorded snapshots of the calculated trajectory. Hydrogen bonds were measured following the native Maestro parameters of a maximum distance of 2.8 Å, a donor minimum angle of 120° and an acceptor minimum angle of 90°. The averages were calculated using the number of broken hydrogen bonds across the entire simulation.

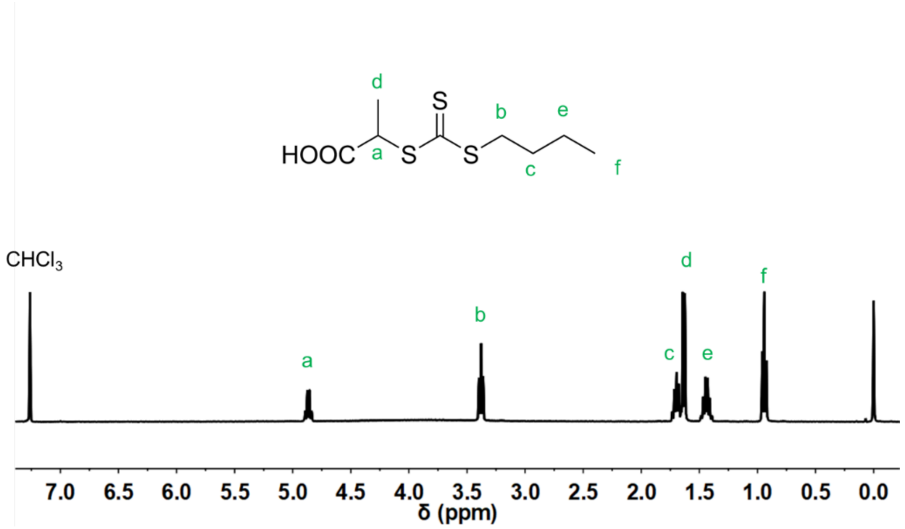

Figure S1. ^{1}H NMR spectrum of PABTC ($CDCl_3$, 25 °C, 400 MHz).

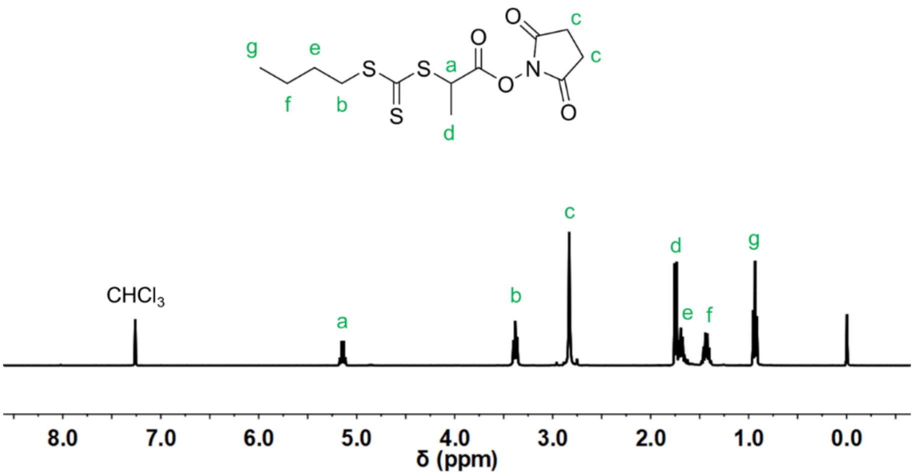

Figure S2. ^{1}H NMR spectrum of PABTC-NHS ($CDCl_3$, 25 °C, 400 MHz).

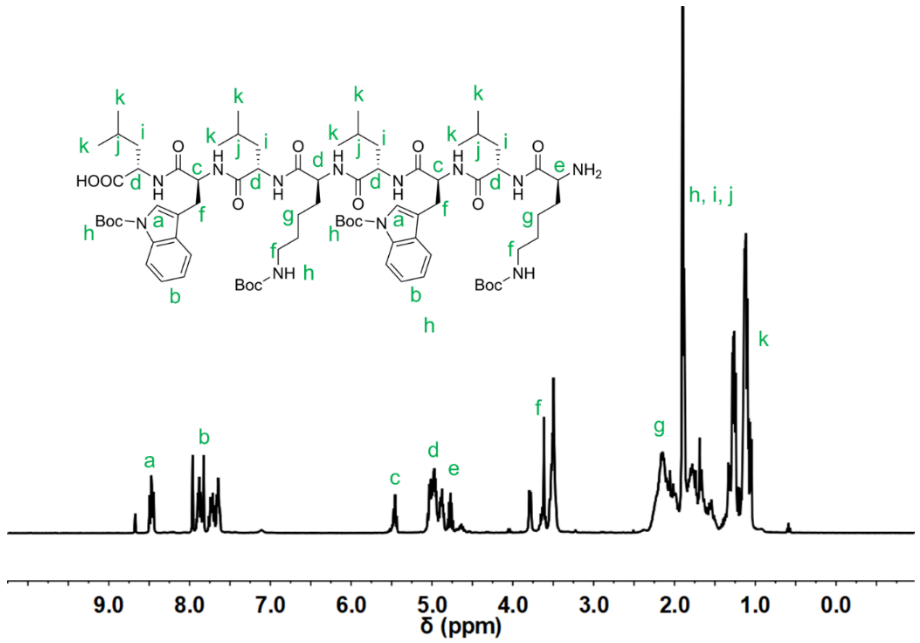

Figure S3. ^{1}H NMR spectrum of linear peptide, LP (TFA-*d*, 25 °C, 400 MHz).

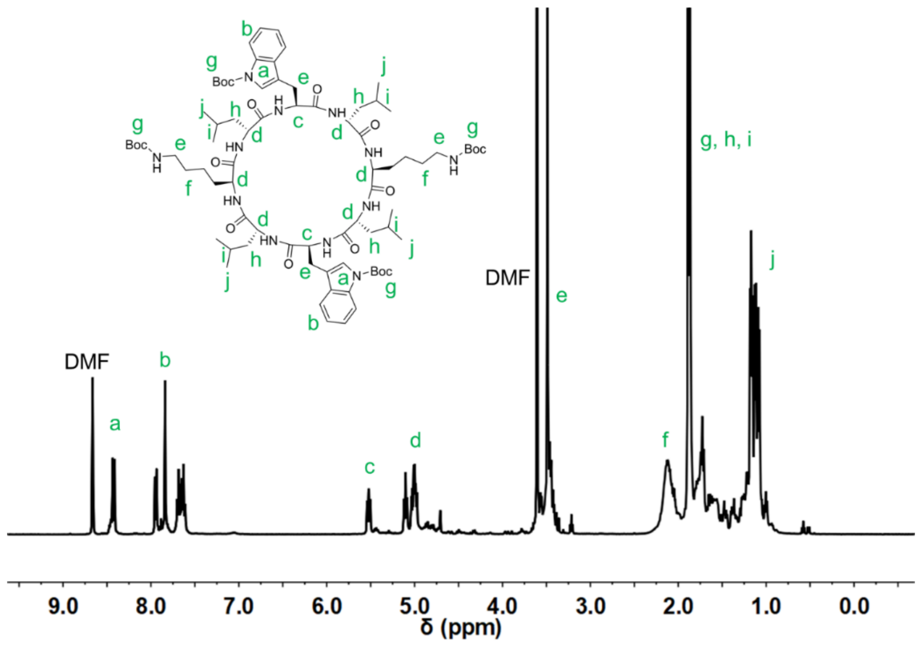

Figure S4. ^{1}H NMR spectrum of BocHN-CP-NHBoc (TFA-*d*, 25 °C, 400 MHz).

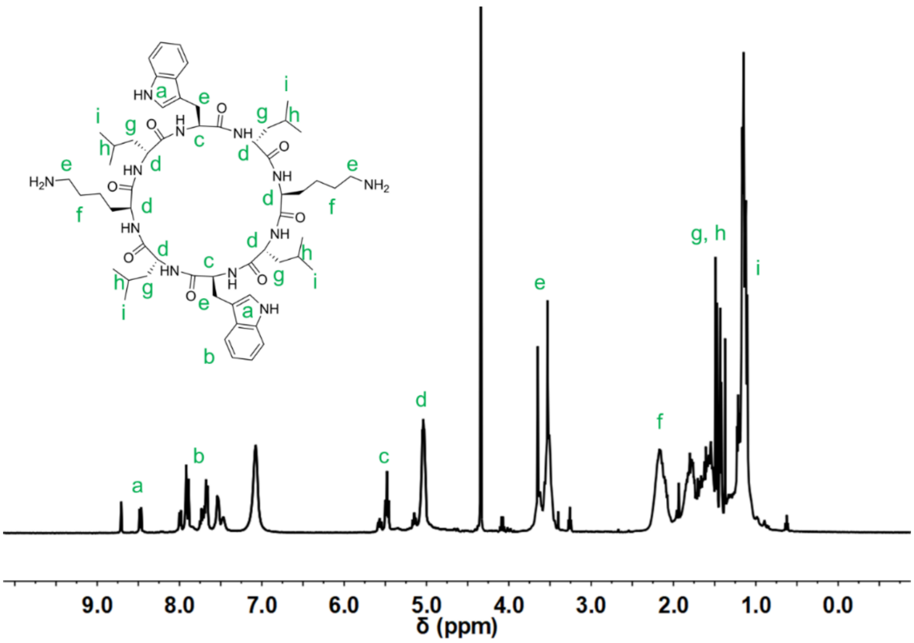

Figure S5. ^{1}H NMR spectrum of H_2N-CP-NH_2 (TFA-*d*, 25 °C, 400 MHz).

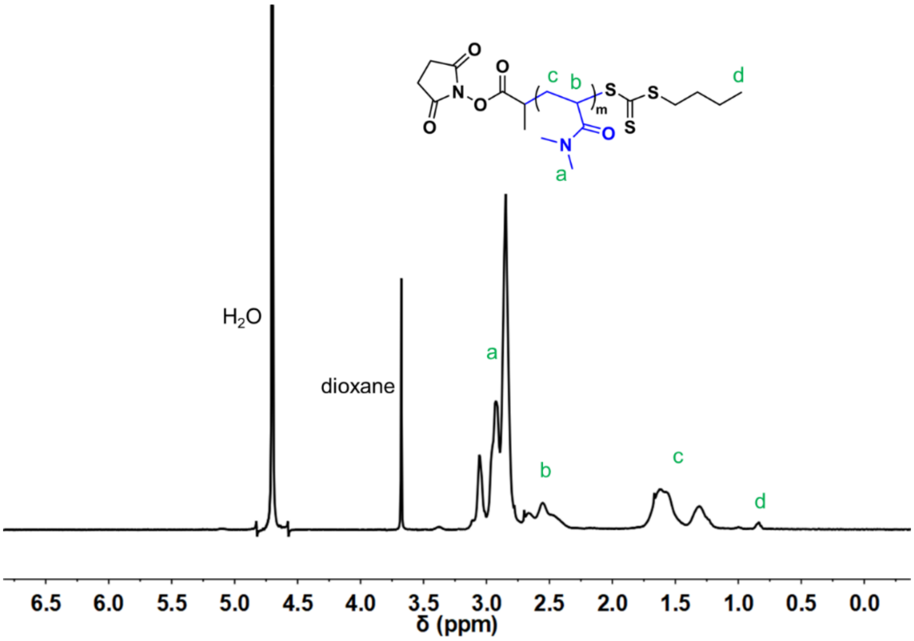

Figure S6. ^{1}H NMR spectrum of PDMA-NHS (D_2O, 25 °C, 400 MHz). The degree of polymerization (DP) of PDMA was measured by using the integration of green 'd' peak (which refers to the -$CH_2CH_2CH_2C\underline{H}_3$, the integration is set as 3.00) and the integration of green 'c' peak (which refers to the backbone -$C\underline{H}_2$CH-, and the integration is 90.18).

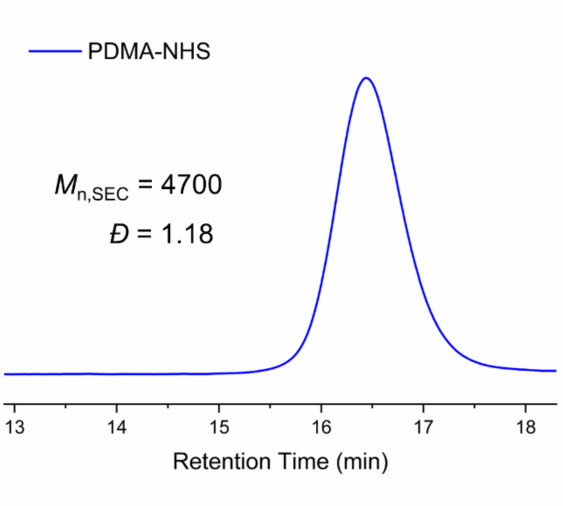

Figure S7. SEC trace of PDMA-NHS (in DMF/LiBr).

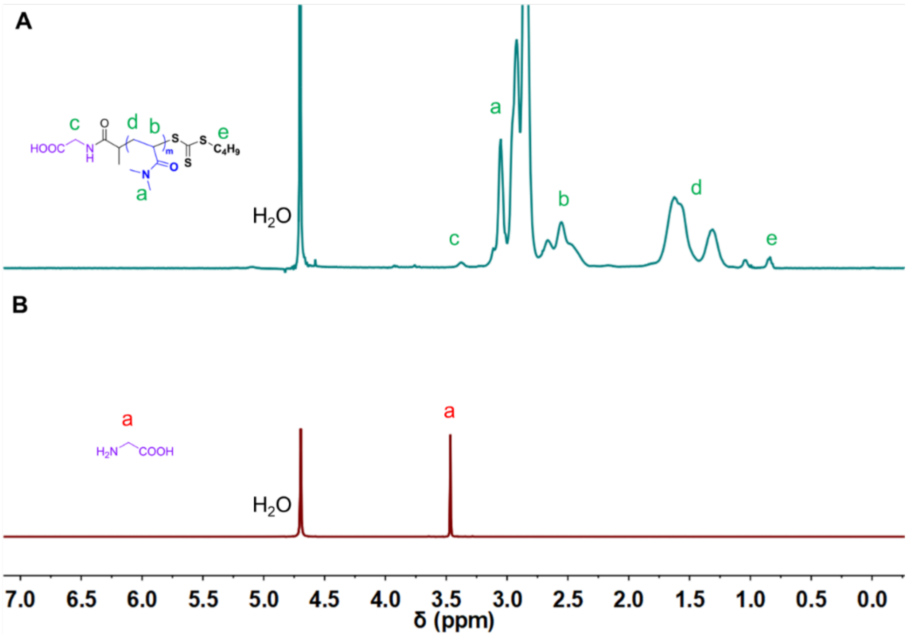

Figure S8. ^1H NMR spectrum of A) PDMA-C2 and B) glycine (C2) (D_2O, 25 °C, 400 MHz).

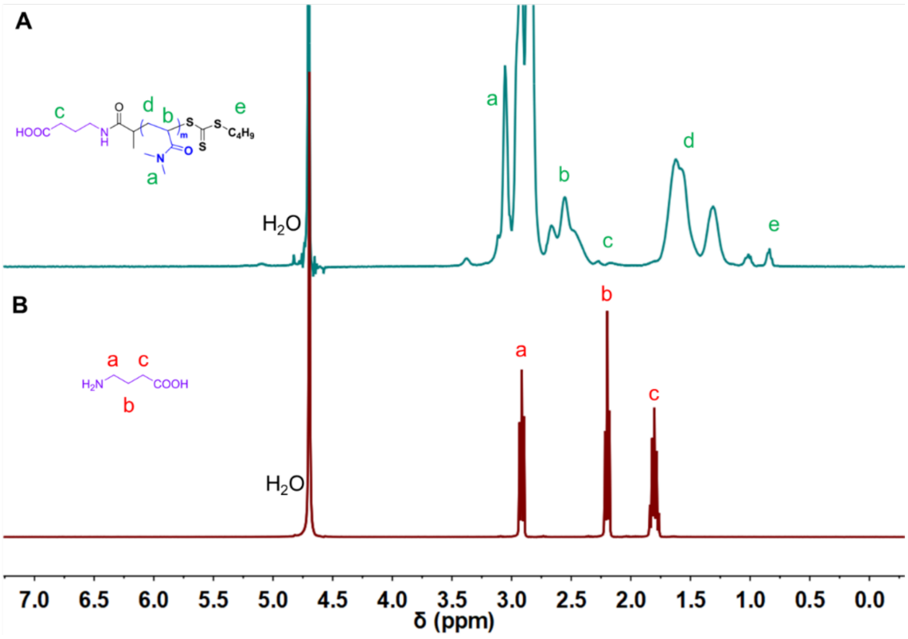

Figure S9. ^{1}H NMR spectrum of A) PDMA-C4 and B) 4-aminobutyric acid (C4) (D_2O, 25 °C, 400 MHz).

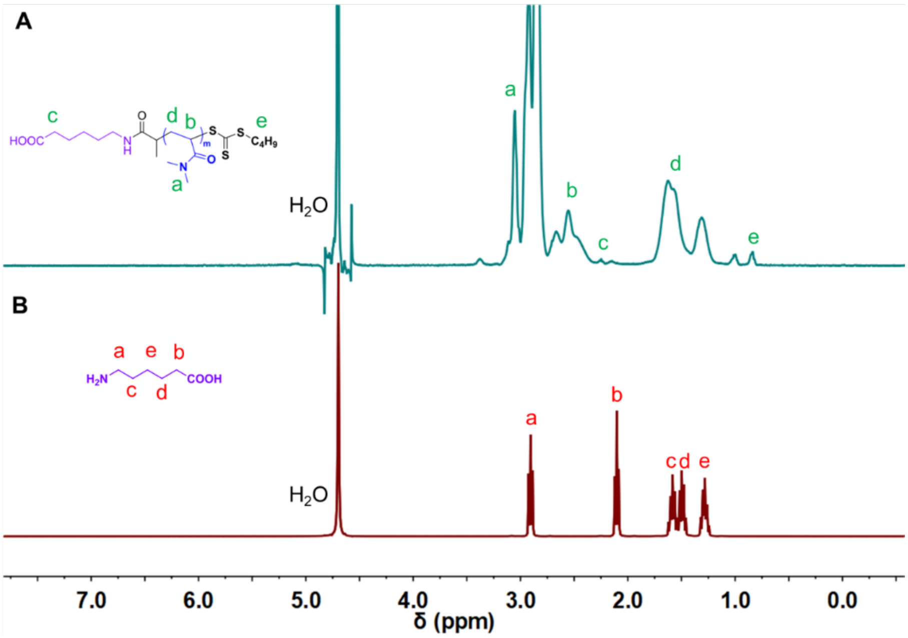

Figure S10. ^{1}H NMR spectrum of A) PDMA-C6 and B) 6-aminocaproic acid (C6) (D_2O, 25 °C, 400 MHz). The end group functionality was measured by using the integration of green 'c' peak (which refers to the HOOCC$\underline{H}_2$-, the integration is 1.97) and the integration of green 'e' peak (which refers to the -$CH_2CH_2CH_2C\underline{H}_3$, and the integration is set as 3.00).

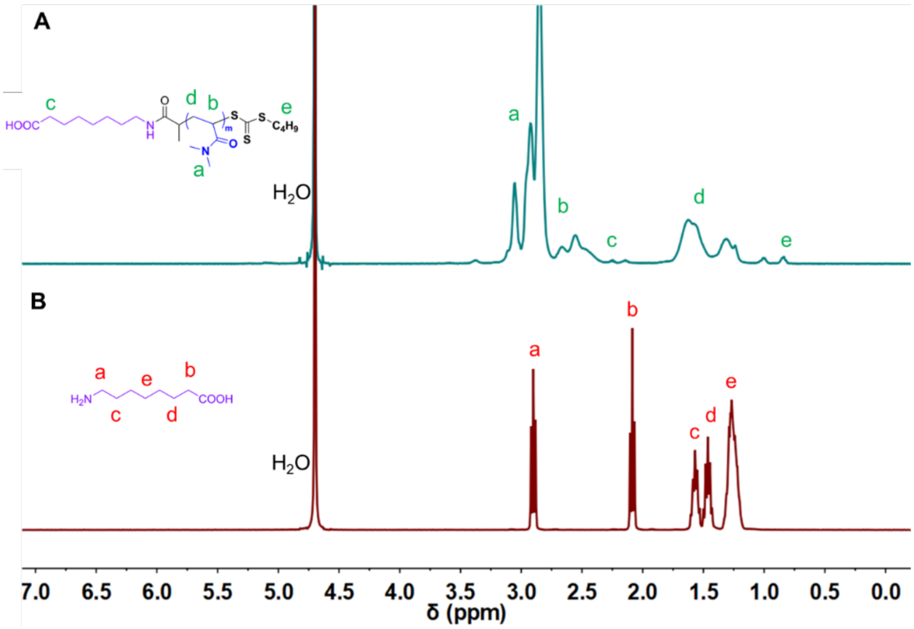

Figure S11. ^{1}H NMR spectrum of A) PDMA-C8 and B) 8-aminocaprylic acid (C8) (D_2O, 25 °C, 400 MHz).

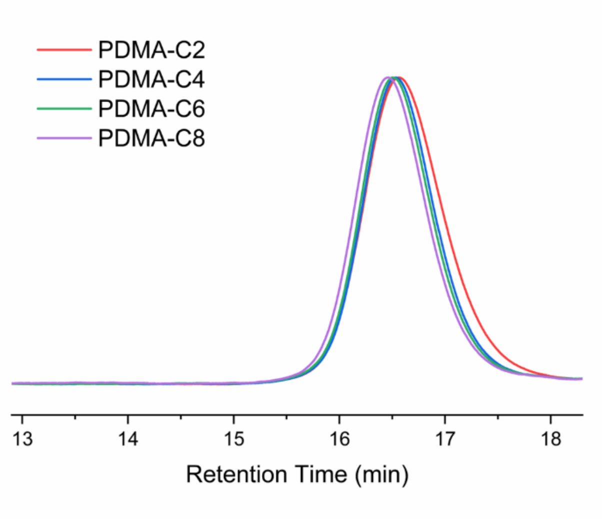

Figure S12. SEC traces of hydrophilic polymer with attached linker: PDMA-C2, PDMA-C4, PDMA-C6, and PDMA-C8 (in DMF/LiBr).

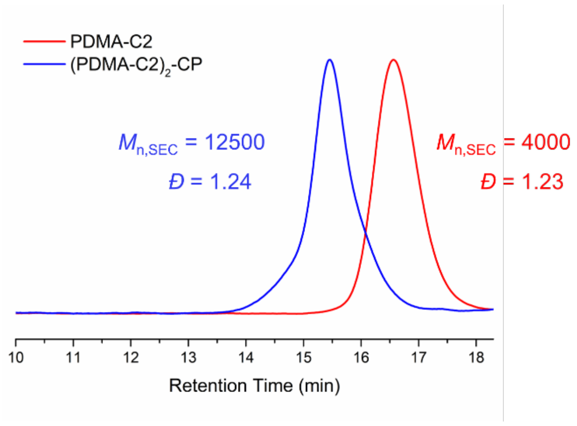

Figure S13. SEC traces of SEC traces of PDMA-C2 and (PDMA-C2)$_2$-CP (in DMF/LiBr).

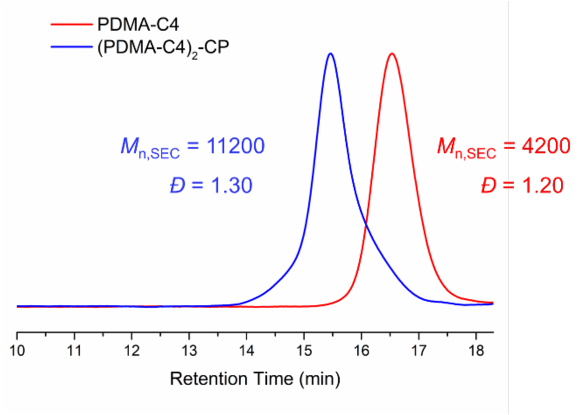

Figure S14. SEC traces of SEC traces of PDMA-C4 and (PDMA-C4)$_2$-CP (in DMF/LiBr).

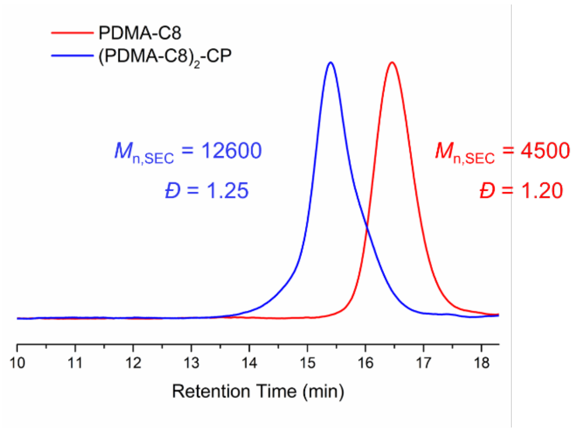

Figure S15. SEC traces of SEC traces of PDMA-C8 and (PDMA-C8)$_2$-CP (in DMF/LiBr).

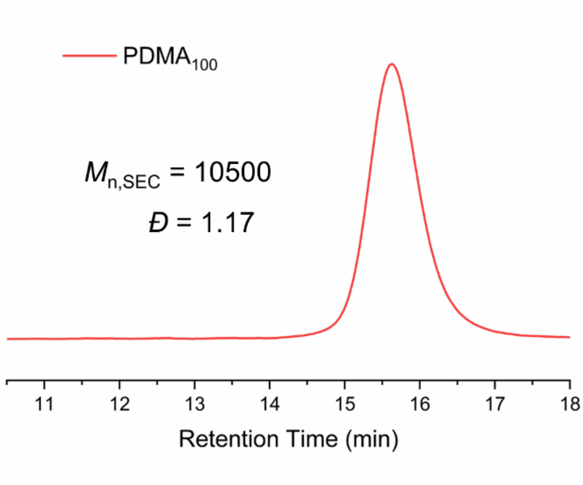

Figure S16. SEC trace of $PDMA_{100}$ (in DMF/LiBr).

Table S1. Fitting parameters using a core-shell cylinder model implemented with SasView software

**Parameter**	**Value**
scale	$9.2616e^{-5}$
Background/cm^{-1}	$4.8878e^{-4}$
sld_core/ Å^{-2}	1.4220
sld_shell/ Å^{-2}	0.8607
sld-solvent/ Å^{-2}	6.3901
radius/ Å	17.445
length/ Å	167.05
Fitting error (χ^2)	2.2696

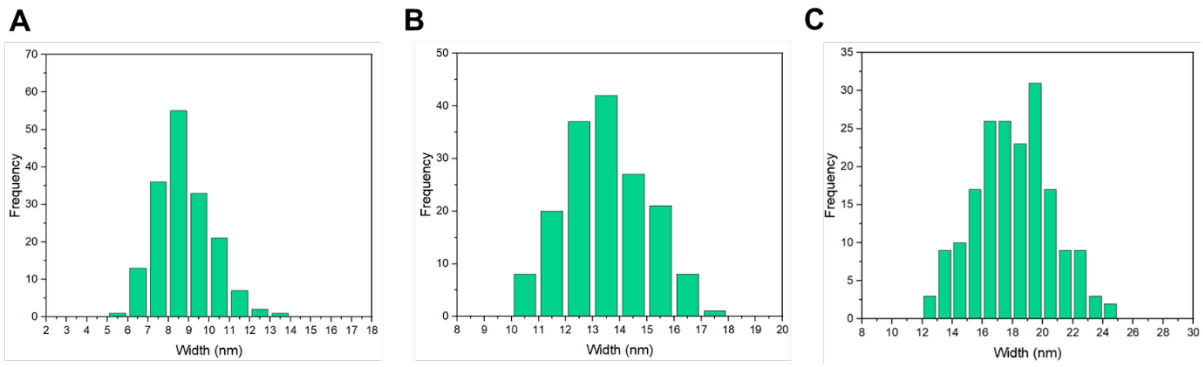

Figure S17. The calculated average width of the nanotubes from TEM images: A) $(PDMA\text{-}C4)_2$-CP, B) $(PDMA\text{-}C6)_2$-CP and C) $(PDMA\text{-}C8)_2$-CP.

Table S2. The correlation of log P value and length of the nanotube.

Sample	Log P[a]	Length (nm)[b]
$(PDMA\text{-}C2)_2$-CP	-0.970	-
$(PDMA\text{-}C4)_2$-CP	-0.190	75
$(PDMA\text{-}C6)_2$-CP	0.590	105
$(PDMA\text{-}C8)_2$-CP	1.370	157

[a] Log P is the octanol-water partition coefficient (log P) value of the linker.

[b] Average length of the nanotube.

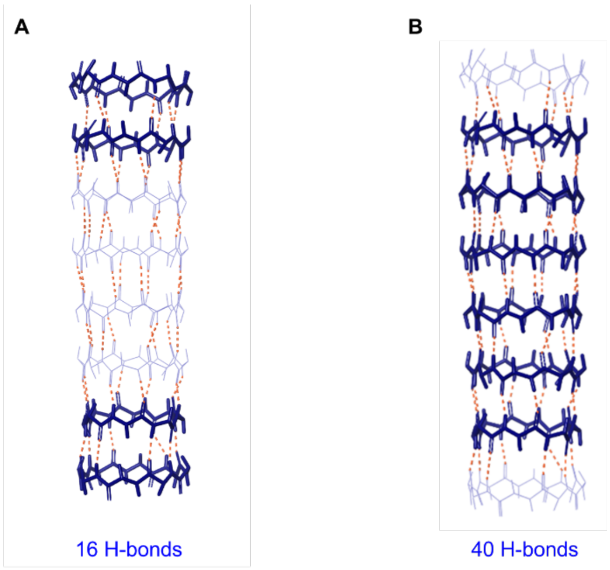

Figure S18. A) Capping and B) core interactions shown as the number of hydrogen bonds formed in the preassembled octameric nanotubes.

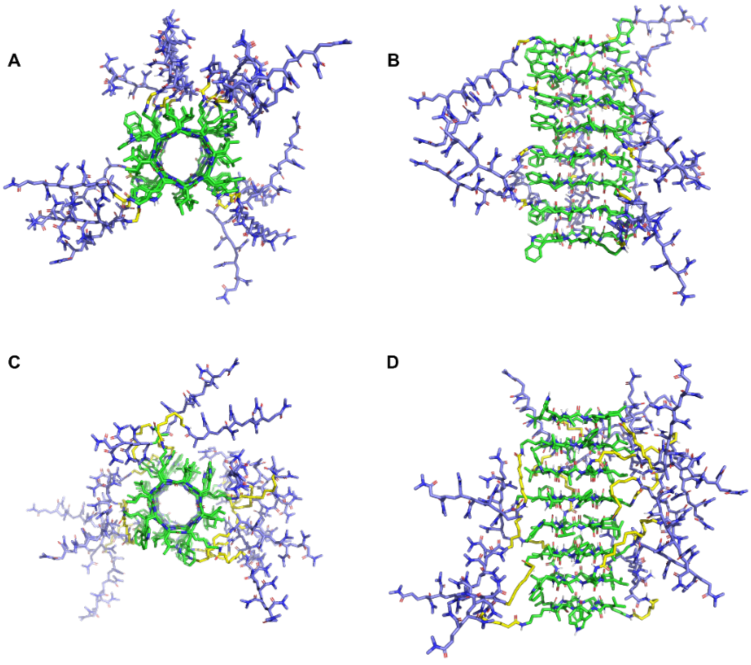

Figure S19. A) Top down, B) side on view of the assembled nanotube system of $(PDMA_6\text{-}C2)_2$-CP. C) Top down, D) side on view of the assembled nanotube system of $(PDMA_6\text{-}C8)_2$-CP. (Backbone: green, C_x: yellow, $PDMA_6$: blue)

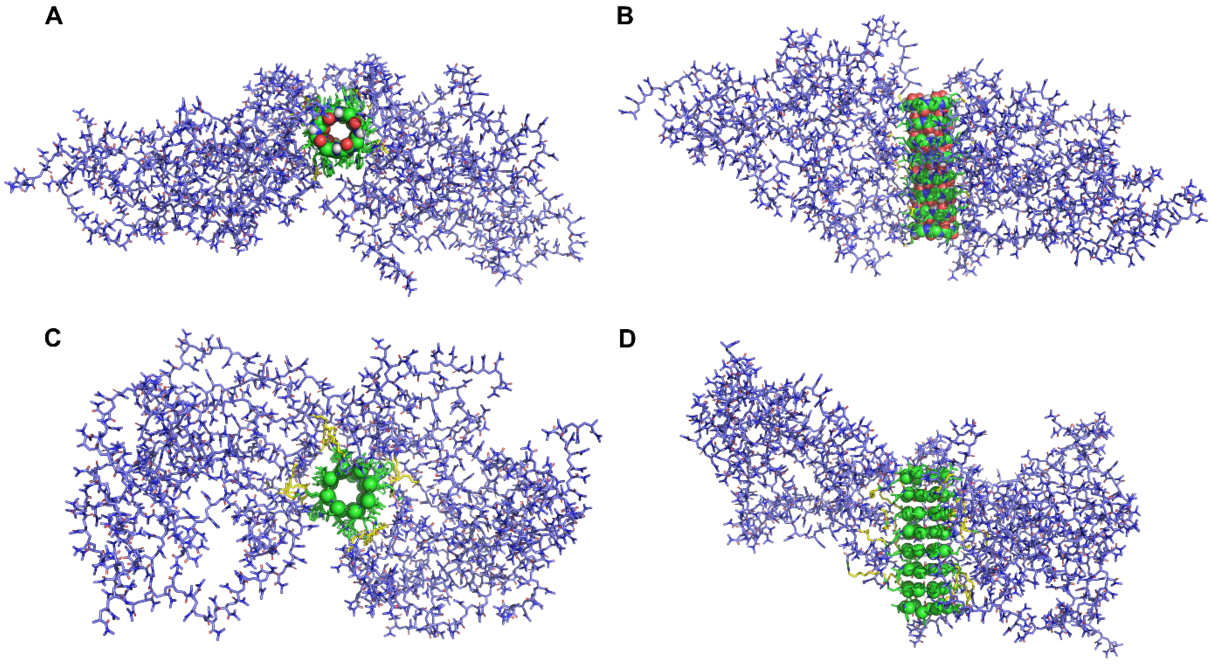

Figure S20. Structure of the assembled nanotube system from a A) top-down view ($(PDMA_{50}$-$C2)_2$-CP, B) side view and C) a top-down view of $(PDMA_{50}$-$C8)_2$-CP and D) side view. (Backbone: green, C_x: yellow, $PDMA_{50}$: blue)

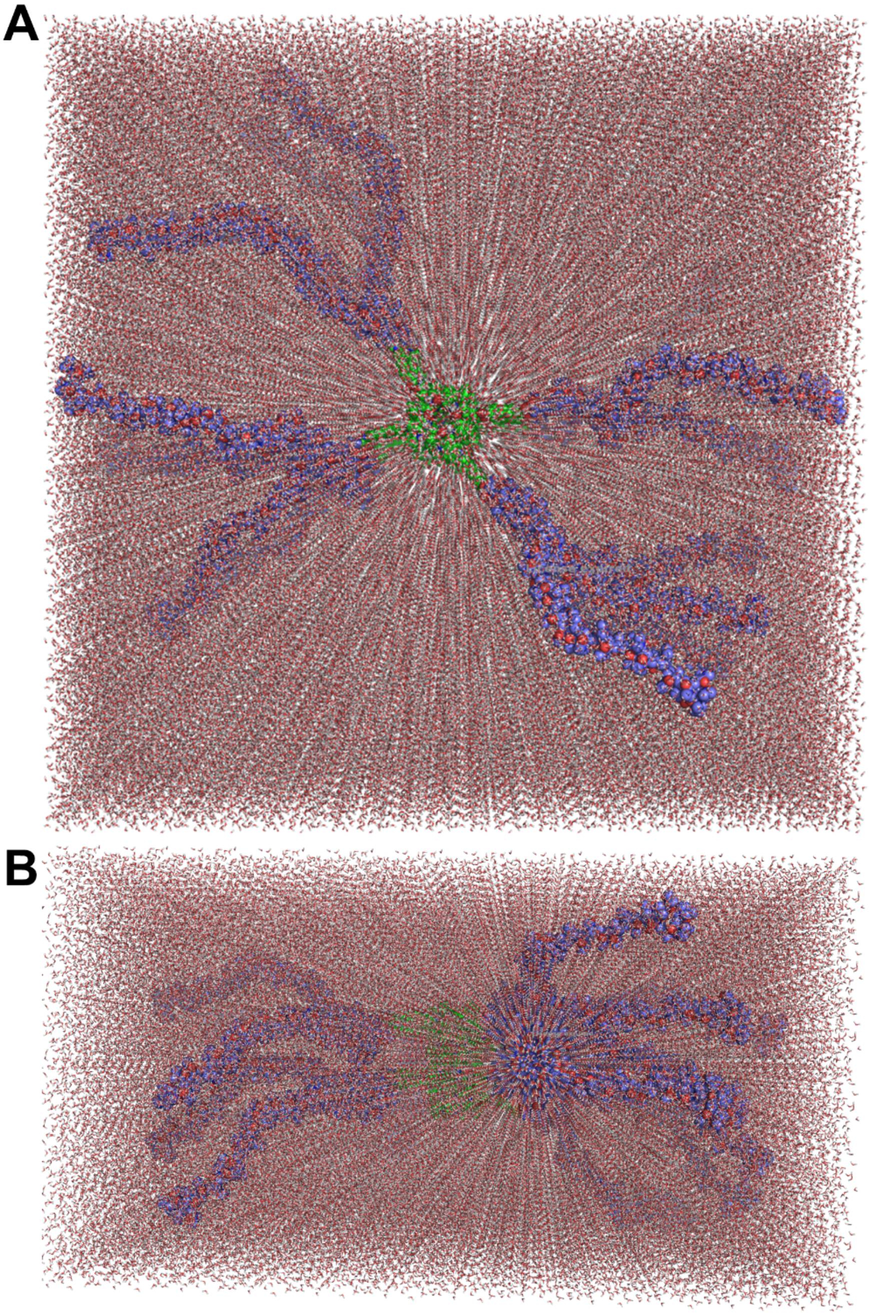

Figure S21. Full system of ($PDMA_{50}$-C8)$_2$-CP solvated in water box, ~570,000 total atoms. A) Top down, B) Side on. (Water: red, C_8-CP: green, $PDMA_{50}$: blue).

References

(1) Raw, S. A. An improved process for the synthesis of DMTMM-based coupling reagents. *Tetrahedron Lett.* **2009**, *50* (8), 946-948.
(2) Lu, C.; Wu, C.; Ghoreishi, D.; Chen, W.; Wang, L.; Damm, W.; Ross, G. A.; Dahlgren, M. K.; Russell, E.; Von Bargen, C. D.; et al. OPLS4: Improving Force Field Accuracy on Challenging Regimes of Chemical Space. *J. Chem. Theory Comput.* **2021**, *17* (7), 4291-4300.
(3) Silk, M. R.; Newman, J.; Ratcliffe, J. C.; White, J. F.; Caradoc-Davies, T.; Price, J. R.; Perrier, S.; Thompson, P. E.; Chalmers, D. K. Parallel and antiparallel cyclic d/l peptide nanotubes. *Chem. Commun.* **2017**, *53* (49), 6613-6616.
(4) Martyna, G. J.; Klein, M. L.; Tuckerman, M. Nosé–Hoover chains: The canonical ensemble via continuous dynamics. *J. Chem. Phys.* **1992**, *97* (4), 2635-2643.
(5) Martyna, G. J.; Tobias, D. J.; Klein, M. L. Constant pressure molecular dynamics algorithms. *J. Chem. Phys.* **1994**, *101* (5), 4177-4189.
(6) Darden, T.; York, D.; Pedersen, L. Particle mesh Ewald: An N·log(N) method for Ewald sums in large systems. *J. Chem. Phys.* **1993**, *98* (12), 10089-10092.
